# Multiple losses of aKRAB from PRDM9 coincide with a teleost-specific intron size distribution

**DOI:** 10.1186/s12915-024-02059-w

**Published:** 2024-11-27

**Authors:** Ann-Christin Zinner, Lars Martin Jakt

**Affiliations:** Faculty of Biosciences and Aquaculture, Nord University, Universitetsalléen 11, Bodø, 8026 Norway

**Keywords:** Meiotic recombination, Teleosts, Intron length, Genome evolution

## Abstract

**Background:**

Primary transcripts are largely comprised of intronic sequences that are excised and discarded shortly after synthesis. In vertebrates, the shape of the intron size distribution is largely constant; however, most teleost fish have a diverged log-bimodal ‘teleost distribution’ (TD) that is seen only in teleosts. How the TD evolved and to what extent this was affected by adaptative or non-adaptive mechanisms is unknown.

**Results:**

Here, we show that the TD has evolved independently at least six times and that its appearance is linked to the loss of the aKRAB domain from PRDM9. We determined intron size distributions and identified PRDM9 orthologues from annotated genomes in addition to scanning 1193 teleost assemblies for the aKRAB domain. We show that a diverged form of PRDM9 ($\beta$) is predominant in teleosts whereas the $\alpha$ version is absent from most species. Only a subset of PRDM9-$\alpha$ proteins contain aKRAB, and hence, it is present only in a small number of teleost lineages. Almost all lineages lacking aKRAB (but no species with) had TDs.

**Conclusions:**

In mammals, PRDM9 defines the sites of meiotic recombination through a mechanism that increases structural variance and depends on aKRAB. The loss of aKRAB is likely to have shifted the locations of both recombination and structural variance hotspots. Our observations suggest that the TD evolved as a side-effect of these changes and link recombination to the evolution of intron size illustrating how genome architectures can evolve in the absence of selection.

**Supplementary Information:**

The online version contains supplementary material available at 10.1186/s12915-024-02059-w.

## Background

In eukaryotes, the coding regions of genes are typically interrupted by segments termed introns [1–3]. During pre-mRNA splicing, expressed sequences (exons) are joined together to form mature mRNA, whereas most released intronic RNAs are rapidly degraded [4]. In most eukaryotes, introns comprise the majority of the primary transcription product [5], increasing both the energetic burden on the cell and the potential for errors during transcription. Spliceosomal introns seem to play a role in most stages of eukaryotic gene expression [6], most notably by increasing the functional complexity of the transcriptome by alternative splicing [7] and the regulation of gene expression [8]. Introns may house both regulatory RNA and enhancer elements [9] and are involved in other processes such as nonsense-mediated decay [10]. Despite the emerging importance of introns for eukaryotic genomes, much of their evolution and functions remain unclear [6].

**Fig. 1:**
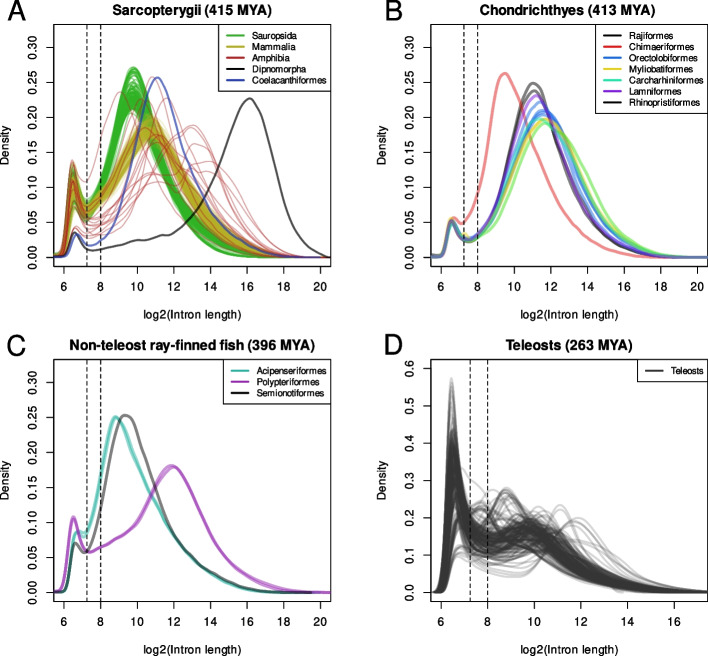
Vertebrate intron size distributions. **A**–**D** Distributions of log transformed intron sizes in (**A**) Sarcopterygii, (**B**) Chondrichthyes, (**C**) non-teleost ray-finned fish and (**D**) Teleostei. Almost all distributions display bimodality, with a distinct peak of short and long introns. The antimode separating these peaks is found at approximately $2^{7.25} (\sim 150)$ bp in non-teleost vertebrates. In contrast the distributions in teleosts are much more varied and the dominant antimode has shifted to $2^8(\sim 256)$ bp or larger. The vertical lines at 7.25 and 8 marks the typical vertebrate and teleost specific antimodes respectively. Numbers in brackets in panel headings indicate the time since the last common ancestor of the species represented in each panel in millions of years ago (MYA). Divergence times were obtained from the TimeTree database [13]

Intron size varies extensively between species and is correlated with genome size in animals [5, 11]. This relationship suggests that introns and intergenic sequences evolve by similar processes and that the study of how intron size evolves can help to explain how genome size evolves. Intron size within genomes vary exponentially [12] (Fig. 1), presumably as a consequence of the likelihood of a structural mutation occurring within an intron being a function of its size. The shape of log transformed intron size distributions across the vertebrates is largely constant; almost all distributions have a small peak of short introns that is separated from a main peak of larger introns by an antimode at around $2^{7.25}$ bp. In contrast, most teleosts have clearly bimodal ‘teleost distributions’ (TDs) characterised by an antimode at, or larger than, $2^8$ bp and a major peak of small introns below this antimode [12]. The TD is not observed in all teleosts but it is found in multiple independent lineages that contain the majority of all known teleost species. Intron and genome size appear to have decreased during teleost evolution [12], and it is possible that the TD has resulted from the retention of functional intron sequences; alternatively, the TD may have evolved as the result of some molecular innovation affecting intron size evolution.

Several studies from the late 90s and in 2000 reported an association of large introns with regions of low meiotic recombination and proposed natural selection to be the driving force for it [14, 15]. In contrast, Nam and Ellegren [16] suggested that the deletion-promoting tendency of recombination itself drives the contraction of introns. In vertebrates, plants and yeast, the majority of recombination events are not randomly distributed along the genome; instead, they are concentrated in narrow regions, known as recombination hotspots [17–21].

In mice, humans [22–24] and probably most other mammals [25–29], the locations of hotspots are determined by the DNA-binding protein PRDM9. In mammals, PRDM9 orthologues contain an ancestral Krüppel-associated box (aKRAB) domain followed by an SSX repression domain (SSXRD), a PR-SET and a series of C2H2 zinc finger (Zn finger) domains [30]. While most vertebrate lineages contain PRDM9 orthologues with all four domains, the N-terminal domains (aKRAB and SSXRD) are missing in several lineages (e.g, teleost fish), and PRDM9 is missing completely (e.g. avians) or pseudogenised (e.g. canids) in others [31, 32].

PRDM9 binds DNA through an array of highly polymorphic C2H2 zinc finger domains leading to trimethylation of H3 lysine 4 and 36 (H3K4Me3, H3K36Me3) through the methyltransferase activity of the PR-SET domain [33, 34]. This leads to the recruitment of the SPO11 endonuclease that catalyses the formation of double-strand breaks (DSBs) that are resolved either by crossover (recombination) or DSB-repair leading to gene conversion [35]. The precise functions of the aKRAB and SSXRD domains are yet to be fully resolved. The aKRAB domain can interact with several proteins that have known roles in meiotic recombination and this is thought to lead to the recruitment of PRDM9-bound DNA to the DSB machinery on the chromosomal axis [36–38].

Since the aKRAB domain has been shown to be required for the function of PRDM9 in mice [36, 37], its absence is likely to indicate a change in PRDM9 function. In avian species (that completely lack PRDM9), recombination events appear to be concentrated in functional regions of the genome, such as promoter-like regions [39]. However, the two mechanisms do not seem to be mutually exclusive, as recombination events in the corn snake are directed to both functional regions and PRDM9 binding sites [40].

We were surprised to note that a number of teleost species reported to have retained a complete PRDM9 (e.g. *Esox lucius, Gadus morhua*, *Clupea harengus* and *Hucho hucho*; Additional file 2 in [12]) did not display the TD. In contrast, species where PRDM9 lacked the aKRAB domain appeared to have the TD. Although it has been shown that PRDM9 lacks aKRAB in a number of teleosts species, it is not clear where and how many times the aKRAB domain has been lost during teleost evolution nor how many times the TD has evolved. Without this information it is difficult to link the appearance of the TD with the loss of the aKRAB domain from PRDM9.

We have extensively surveyed the teleost lineage both for the presence of the TD and the domain content of PRDM9 orthologues. We identified PRDM9 candidates in almost all annotated genomes, suggesting that most or all teleosts do have PRDM9 orthologues. PRDM9 orthologues could be divided into two main classes, PRDM9-$\alpha$ and PRDM9-$\beta$, in line with a previous classification [31]. The $\beta$ type was found in almost all species analysed; in contrast, the $\alpha$ type was absent from all Euacanthomorphacea, and in these species, we observed diverged PR-SET domains suggesting neofunctionalisation. Most species outside the Euacanthomorphacea contained both types of PRDM9, but in most cases, aKRAB was absent from PRDM9-$\alpha$. We demonstrate that aKRAB has been lost from PRDM9 multiple times independently and show that the TD is found only in species that lack aKRAB. This argues that loss of the aKRAB domain from PRDM9 represents a molecular innovation that has contributed to the appearance of the TD. We note that this implies that the TD may have evolved in parallel in a number of lineages in the absence of selection.

## Results

### The TD has evolved multiple times during teleost evolution

To determine the number of times the TD has evolved within the teleosts, we extended our previous analysis to include species from all major teleost clades. To this end, we examined intron size distributions of 216 teleost species where annotation was available from NCBI.

The distributions were highly variable, but in almost all species we observed a notable peak of minimally sized introns and a broad peak of longer introns. In the majority of species the peak of small introns was markedly higher and the antimode was around $2^8$ bases (up to $2^9$ in a small number of species) with a distribution fitting the TD (Fig. S1, Additional file 1). Distributions that did not fit the TD generally had lower and narrower peaks of small introns, reminiscent of those observed in non-teleost vertebrates (Fig. 1).

**Fig. 2:**
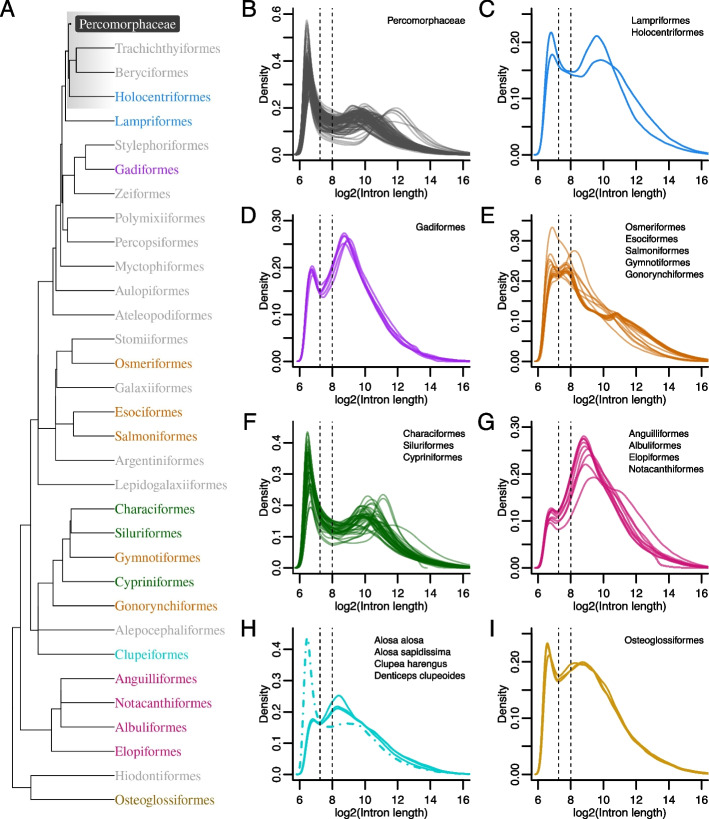
Teleost phylogeny and associated intron sizes. **A** Phylogenetic tree of teleost orders obtained from the TimeTree database [13] with the percomorph clade collapsed. Colours are those used for plots of intron size distributions in (**B**–**I**). The clade Euacanthomorphacea is highlighted in grey. **B**–**I** Log transformed intron size distributions of species belonging to clades indicated by colours in (**A**). Note that panels (**C**, **E** and **F**) contain distributions from multiple clades and do not represent monophyletic groups. Vertical lines at 7.25 and 8 mark the positions of the antimodes seen in most non-teleost vertebrates and in teleosts respectively

We mapped the species to a phylogenetic tree of teleost orders obtained from TimeTree [13] in order to assess the taxonomic distribution of the TD (Fig. 2). The majority of the species with distributions resembling the TD are percomorphs (clade Percomorphaceae) which include more than half of extant teleosts [41]. Other species with this distribution were spread across the teleost tree in pattern suggesting that the TD has evolved independently at least 4 times in (1) the lineage leading to *Denticeps clupeoides* (Fig. 1H) of the order Clupeiformes, (2) the order Cypriniformes (Fig. 1F), in the most recent common ancestors of (3) the Siluriformes and Characiformes (Fig. 1F) and (4) the Euacanthomorphacea and Lampriformes (Fig. 1B, C).

The TD was not present in a number of independent teleost lineages (Fig. 2D, E, G, H, I). These included distributions from gadiform and osteoglossiform species which also had distinctive antimodes; however these were shifted to the left and different from the TD (Fig. 2D, I).

### Distinct domain structures of PRDM9 orthologues

In order to clarify the relationship between the absence of aKRAB in PRDM9 and the presence of the TD, we first set out to establish the typical domain structure of teleost PRDM9 orthologues. To this end, we identified genes containing the term ‘PRDM9’ from 191 annotated teleost genomes and used Interproscan [42] to determine their domain content. Of these species, 162 contained 378 genes annotated with PRDM9. Most species contained one or two candidate orthologues, but siluriform species typically had larger numbers with one species (*Clarias magus*) having 34 candidates (Fig. S2, Additional file 2).

Most orthologue candidates contained both a PR-SET and multiple Zn finger domains but lacked the aKRAB domain (Fig. S3, Additional file 2) consistent with previous observations [32]. We considered genes as PRDM9 orthologues only if the corresponding protein sequences had a PR-SET domain and discarded candidates without PR-SET from further analyses.

Intron positions within sequences encoding the aKRAB and PR-SET domains (henceforth, simply domains or sequences) were generally conserved, but the sequences between the two domains contained a variable number of exons of different sizes (Fig. S3, Additional file 2). Remarkably, the C-terminal Zn finger domains and interspersing sequences contained almost no introns in any of the genes. The PR-SET domain was contained within three exons of similar sizes to those in mammalian orthologues in almost all sequences; however, we observed an additional intron in the PR-SET domain of three of four PRDM9 orthologues in *G. morhua*, suggesting a recent intron insertion in the Gadiformes.

We aligned the amino acid sequences of exons containing the PR-SET domain and flanking regions to explore the relationship between the PR-SET sequence and domain organisation. The organisation of the Zn fingers correlated with the clustering of the PR-SET domain (Fig. S4, Additional file 2). Notably, most proteins containing an aKRAB domain had a common Zn finger domain structure, with an optional proximal Zn finger separated from a single terminal array of Zn fingers, as reported for mammalian PRDM9 orthologues [43]. An analogous Zn finger organisation was also present in a large number of sequences that lacked the aKRAB domain but whose PR-SET domain sequences clustered with those from aKRAB containing proteins.

### An HMM recognising an extended PRDM9 PR-SET domain

We made use of the multiple sequence alignment of the extended PR-SET sequences to define a hidden Markov model (ePR-SET). We used this, along with an HMM developed specifically against the aKRAB domain (aKRAB) [44] as well as Prosite SET (PS50280, SET), KRAB related (PS50806, KRAB-R) and C2H2 Zn finger (PS50157, PS-ZNF) models, to scan protein sequences from 185 teleost species in order to identify additional PRDM9 orthologues.

As expected, the results were dominated by matches to the Zn finger domains ($>10^5$ proteins), followed by matches to the PR-SET domain models ($\sim 10^4$ proteins). In contrast, we observed relatively few matches to the aKRAB and KRAB-R domains (57 and 44, respectively; Fig. S5, Additional file 2). All matches to KRAB-R (44) were contained in the matches to the aKRAB model and 27 of the matching proteins also contained PR-SET and Zn finger domains. The score distributions for both the ePR-SET and aKRAB HMMs were multimodal and suggested several score thresholds. For the aKRAB HMM, the scores could be divided into three clear regions: 0–20, 38–45 and above 50 (Fig. S6, Additional file 2).

The ePR-SET HMM scores could also be divided into a number of classes with a clear gap in scores between 180 and 250 (Fig. S6, Additional file 2). This distinction was most clearly seen from the relationship between the Prosite SET (PS5028) and the ePR-SET scores which divided the proteins into a number of distinct groups (Fig. S7, Additional file 2). Notably, all protein sequences containing matches to aKRAB and SET models had ePR-SET scores above 180. We also scanned the mouse, human and 379 avian proteomes for the ePR-SET model. In mouse and humans, we observed three different groups of well separated scores: those for PRDM9 and PRDM7 ($\sim 230$), PRDM11 ($\sim 180$) and all others ($<102$) (Fig. S8, Additional file 2). In avians, no proteins obtained scores above 186.7 (Fig. S9, Additional file 2). Taken together, this argues that our HMM model is highly specific for vertebrate PRDM9 orthologues.

We obtained scores for the ePR-SET HMM above 180 in 180 of the 185 species analysed. However, considering only canonical proteins (one per gene) that also were recognised by the Prosite SET model, we identified candidate orthologues from 176 species that were further analysed. In most species, we detected no more than two matching proteins, but in several species from related clades, we found numerous orthologue candidates (Fig. S10, S11). Again, most of the gene expansion appears to have occured in Siluriform species. In addition, *Aldrovandia affinis* (order Notacanthiformes) contained a number of proteins with both aKRAB and ePR-SET matches; however, most of these appeared truncated with no, or only one Zn finger.

### Distinct classes of PRDM9 PR-SET domains

**Fig. 3:**
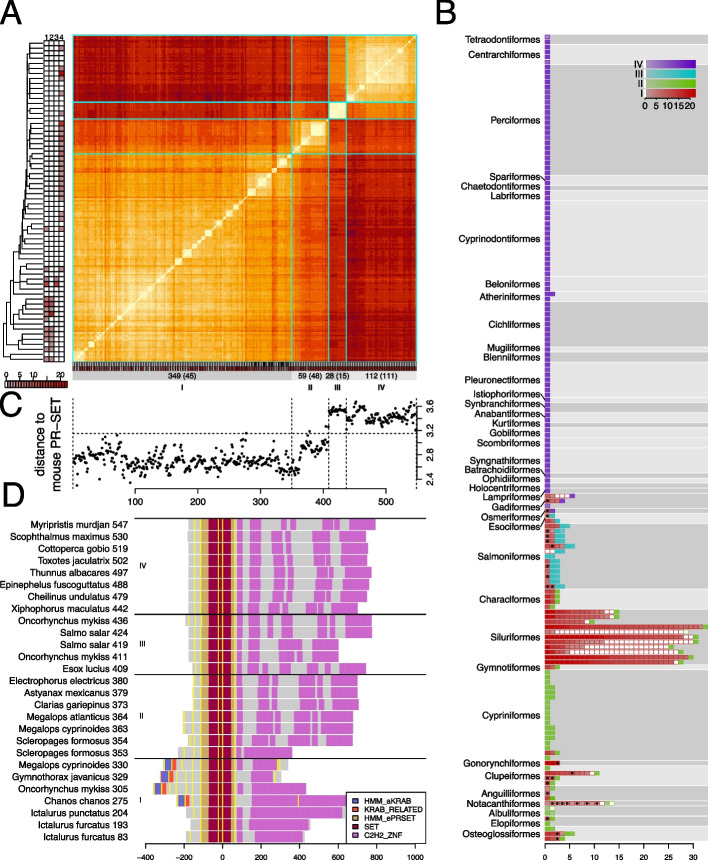
Distinct classes of PR-SET domains in teleost PRDM9 orthologues. Sequences matching an extended PR-SET model were aligned and pairwise distances calculated. **A** Pairwise distances for 548 teleost ePR-SET sequences. Colours range from light yellow (low) to dark red (high). Rectangles underneath the plot indicate the presence of an aKRAB domain (black) and the number of Zn finger domains identified (red colour scale) in the respective proteins. The sequences were divided into four classes indicated by cyan lines and the panel underneath. Numbers indicate the total number of proteins and (species) for each class. Left panel shows the number of species with matches to the different classes of PR-SET sequences for individual teleost orders. The number of Zn-fingers (below) and number of species (to the left) use a common colour scale truncated to a max of 22 Zn fingers. **B** Number of proteins of each class. Each bar represents a single species, with each rectangle representing a single protein. The outline and internal colours represent the PR-SET class and the number of Zn-finger domains respectively as indicated in the inset. Proteins containing an aKRAB domain are indicated with a black point. **C** Distance (dis-similarity) of teleost ePR-SET sequences to the corresponding mouse sequence. Class I and II sequences are more similar than class III and IV. **D** Domain structures of example proteins with different ePR-SET classes. Class I proteins have a common domain structure with a terminal long Zn-finger array whereas the other classes have multiple short Zn-finger arrays

We performed a multiple sequence alignment of 548 sequences matching the ePR-SET and Prosite SET models and determined pairwise distances for this extended set of orthologue candidates. The sequences divided into four apparent classes (Fig. 3A), only one (I) of which contained proteins with aKRAB domains. Class IV domains were found in the branch of teleosts from the Percomorphs to the Osmeriformes (Euacanthomorphacea, Lampriformes, Gadiformes and Osmeriformes) with exactly one matching protein in all species except *Menidia menidia* which had two. Species with class IV proteins did not contain other classes except in the Gadiformes and Osmeriformes which also contained class I proteins. In contrast, class I proteins were found from the Gadiformes to the Osteoglossiformes and all these species, except *Gymnothorax javanicus* and *Chanos chanos*, also contained proteins with one other domain type (Fig. 3A, B).

The class I and II domains were more similar to the ePR-SET sequence found in mouse than class III and IV (Fig. 3C). This suggested two primary classes of PR-SET domains in the teleosts (I,II and III,IV) that could have arisen from a gene duplication event in a common ancestor of mammals and teleosts. However, the Zn finger domain structures of class I was distinct from that of classes II to IV (Fig. 3D, S12), and the distances between class I and class IV were smaller than those between class IV and the mouse sequence (Fig. S13, Additional file 2). Furthermore, the distance between classes II and III was similar to the distance between classes II and I.

**Fig. 4:**
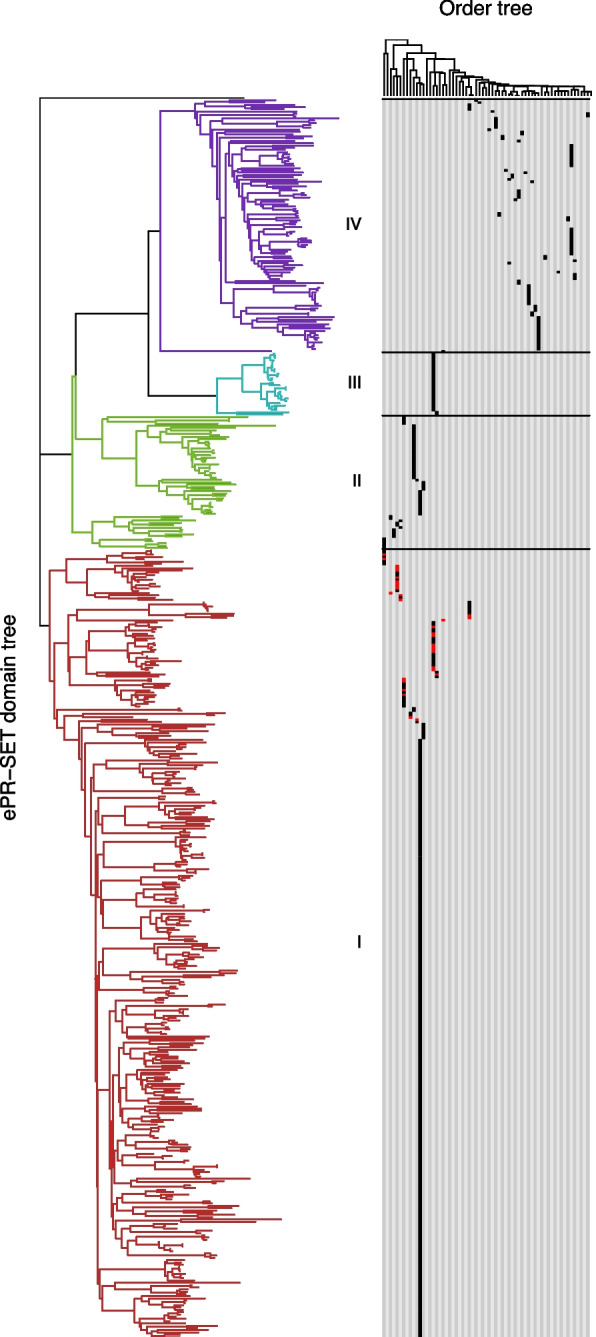
Two types of teleost PRDM9 PR-SET domains. Left: a neighbour-joining tree derived from ePR-SET distances rooted by the mouse ePR-SET sequence. Colours indicate the classes inferred from the distance matrix (Fig. 3A). Right: the taxonomic source of the protein sequences represented in the tree. A phylogenetic tree of teleost orders is shown at the top running left to right from Osteoglossiformes to Lophiiformes. The taxonomic source of proteins is indicated by filled rectangles. Red rectangles indicate proteins that contain the aKRAB domain. The large branch of class I proteins at the bottom are all from the Siluriformes order

These distances were reflected in a neighbour joining tree where classes II to IV formed a monophyletic group separate from class I (Fig. 4). The topology of this tree and the distribution of classes across the teleost orders support the notion that classes II to IV are derived from a single ancestral sequence and have diverged by speciation rather than gene duplication. For example, we do not observe any species that contain a combination of classes II to IV. Hence, our observations are consistent with a previous classification [31] of teleost PRDM9 into an $\alpha$ (class I) that may contain the aKRAB domain and a $\beta$ type (classes II to IV).

Surprisingly, we find that the more conserved of these classes ($\alpha$) has also undergone extensive gene amplification in several lineages, most notably so in the Siluriformes. In contrast, we only find multiple copies of the $\beta$ type in a small number of species, and these may be related to recent genome duplications in the salmonids [45] and cyprinids [46].

Taken together, our results are consistent with previous classification, but indicate an accelerated divergence of PRDM9-β in the evolution of the Euacanthomorphacea lineage.

### Eight potential losses of the aKRAB domain in the teleosts

Our observations indicate that loss of aKRAB has occurred multiple times during teleost evolution; however, the small number of species we identified containing PRDM9 orthologues with aKRAB domains makes it difficult to define at what points the domain has been lost. To address this, we extended our analyses to unannotated genomes by searching six-frame genome translations from 1193 distinct species, representing 60 orders, for matches to the aKRAB HMM used above. This model recognises sequences specifically from a single exon and can thus be used directly against genome sequences without prior gene prediction.

The highest score from each species showed a marked shift in values around 30 (Fig. S14, Additional file 2) corresponding to that seen against protein sequences. Of the 1193 species, 1170 were associated with an NCBI order; all but two of which (Carangiformes and Acropomatiformes) were represented on the TimeTree teleost phylogeny. The species not associated with a taxonomic order were all percomorphs, none of which contained candidate aKRAB domains. As they did not appear informative we excluded them from further analyses.

**Fig. 5:**
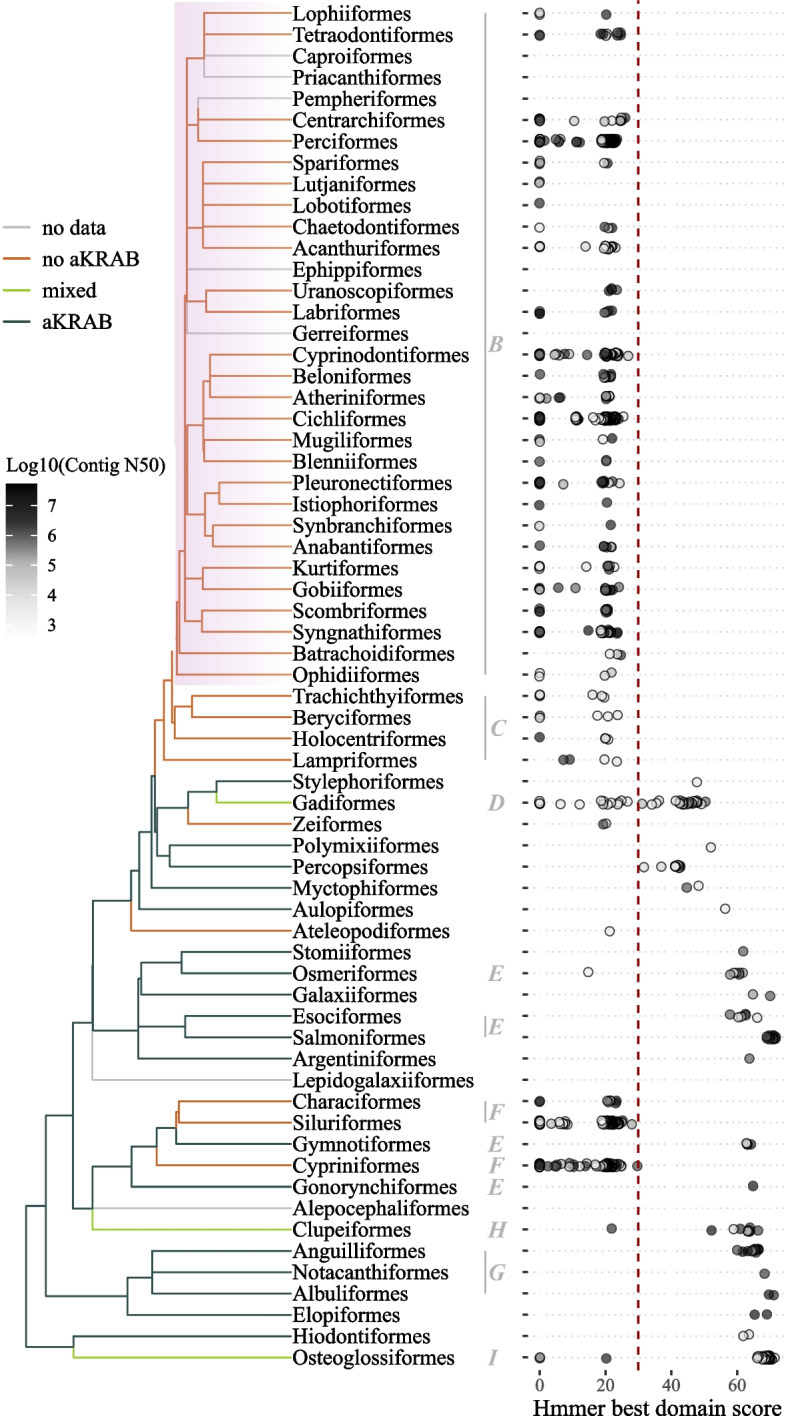
Phylogenetic distribution of ancestral KRAB (aKRAB) domain matches and inference of the location of losses. Left: phylogenetic tree of teleost orders. Right: the single highest hmmer domain score for the aKRAB domain for each species ordered vertically by taxonomic order. The N50 of each assembly is indicated by the shade of grey as shown in the key. The phylogenetic location of losses was inferred by dividing the tree into sets of monophyletic groups where all species lack matches to the aKRAB domain (red branches) and finding their most recent common ancestor. Green lines indicate orders containing a mixture of species with and without matches. The percomorph radiation is highlighted in purple. Red vertical line at a score of 30 represents threshold between absence and presence of aKRAB

As predicted by our initial analyses, we only observed matches to the aKRAB model within the branch of teleosts containing class I PRDM9 proteins (Fig. 5). Six monophyletic groups clearly lacked aKRAB domains; four of these are essentially identical to the ones reported previously [32]: (1) the family Osteoglossidae (Fig. S15, Additional file 2), (2) the Cypriniformes, (3) Characiformes and Siluriformes and (4) Lampriformes and Euacanthomorphacea. We also observed a clear absence in *D. clupeoides* (Fig. S16, Additional file 2), in the order Zeiformes and a likely absence in *Guentherus altivela* (order Ateleopodiformes).

The status of aKRAB in *G. altivela* was unclear. We did not find any candidate loci, but with only one very fragmented assembly available for Atelopodiformes, we could not exclude a false negative. To confirm a potential absence, we performed a tblastn search of the raw reads from *G. altivela* against the N-terminal portion of the *G. morhua* PRDM9 protein sequence. Fewer reads than expected, given the coverage, could be aligned to the N-terminus of the aKRAB domain (Fig. S17, Additional file 2), and no reads at all aligned to the C-terminal part of the aKRAB. These results are consistent with a loss of aKRAB in *G. altivela* but could also be due to less conservation of this aKRAB part or the insertion of an additional intron in this region.

The presence of a complete PRDM9 has been established only for a single species within Gadiformes (*G. morhua*) [32]; however, an aKRAB domain could be identified in 28 of the 46 gadiform assemblies (Fig. S18, Additional file 2). We suspect that most of the absences are due to the low assembly quality of these species. To test this suspicion, we performed tblastn, as done for *G. altivela*, on the raw sequencing reads of nine species from genera where we failed to detect aKRAB in all assemblies. In all but three species (*Bregmaceros cantori*, *Coryphaenoides rupestris*, *Macrourus berglax*), we found the expected number of reads aligning to the sequence (Fig. S17, Additional file 2). *C. rupestris* and *M. berglax* are closely related and in this sampling context form a monophyletic group; hence, the absence in both these species may reflect a single ancestral loss. Our observations thus suggest that most gadiform species have an aKRAB domain but that recent losses may have occurred within a small number of genera.

We failed to detect an aKRAB domain in a total of nine lineages; however, two of these were found in the Gadiformes, where they were scattered around and failures to detect appeared to be correlated with the quality of assemblies. Of the gadiform species, the most convincing evidence for an absence of aKRAB was found in *C. rupestris*; if this is included, then we detected eight independent losses in total.

### Loss of aKRAB from PRDM9 is a prerequisite for the TD

Both the absence of aKRAB and the presence of the TD occur in species that can be grouped into a distinct set of monophyletic groups. If the loss of aKRAB is related to the evolution of the TD, then these groups should coincide.

Formally dividing teleost intron sizes into having or not having the TD is difficult due to the large variability of intron size distributions in the teleosts. However, the TD is characterised by an antimode around $2^8$ bases, and we found that a plot of the distribution density at $2^8$ bp versus any size fraction between $2^{10}$ and $2^{16}$ bp divided teleost introns into two groups that correlated with an intuitive distinction (Fig. S19, Additional file 2).

**Fig. 6:**
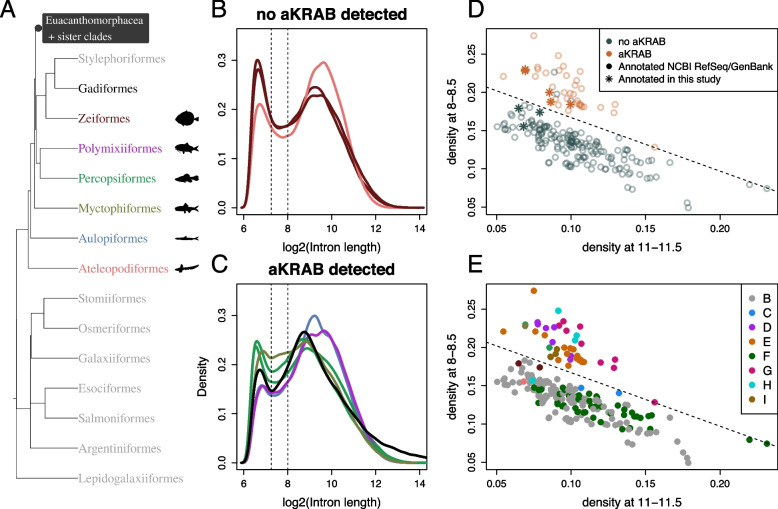
Loss of aKRAB coincides with the TD. **A** Collapsed phylogenetic tree of teleost orders. Orders containing species for which annotation was generated in this study are coloured. **B** $\log _2$ intron size distributions of species where no aKRAB was detected. Colours correspond to labels in (**A**); Zeiformes (*Zeus faber, Cyttopsis rosea*), Ateleopodiformes (*Guentherus altivela*). **C** $\log _2$ intron size distributions of species with aKRAB. Colours correspond to labels in (**A**); Polymixiiformes (*Polymixia japonica*), Percopsiformes (*Percopsis transmontana, Forbesichthys agassizii*), Myctophiformes (*Benthosema glaciale*), Aulopiformes (*Parasudis fraserbrunneri*); the *Gadus morhua* distribution is shown in black. **D**, **E** Plots of distribution densities at the intervals ($2^{11}$–$2^{11.5}$) and ($2^8$–$2^{8.5}$) distinguish distributions from species with and without aKRAB. Points are coloured by inferred aKRAB status (**D**) and taxonomic order (**E**). Distributions from species without aKRAB lie beneath the dividing line (**D**) except for *S. formosus*, *C. rupestris* and *M. polli*. Note that the lack of a match in *M. polli* is either a false negative or represents a recent loss since we did detect a match in *M. productus*. Colours in (**E**) are the same as those in (Fig. 2) and (**A**) and demonstrate the diverse taxonomic origins of both groups

Intron size distributions were available from members of six of the eight monophyletic groups potentially lacking aKRAB; the TD was found in all of these except in *Scleropages formosus* (Osteoglossiformes) and *C. rupestris* (Gadiformes). In contrast, no distributions from species with aKRAB conformed to the TD (Fig. 6). Note that although we did not detect aKRAB in the gadiform *Merluccius polli* by the hmmer scan, aKRAB is likely present or the loss very recent, as we detect it in species of the same genus (Fig. S18, Additional file 2).

Intron size distributions were not available from any members of two orders (Zeiformes, Ateleopodiformes) which appear to lack aKRAB. To test our inference, we estimated intron sizes in members of these orders as well as members of orders where we detected aKRAB (Polymixiiformes, Percopsiformes, Myctophiformes, Aulopiformes) but did not have distributions from close relatives (Fig. 6).

As expected, we observed non-TDs for all species in which we clearly identified aKRAB domains. In contrast, both zeiform species had a distribution that conformed to the TD, in line with the absence of aKRAB (Fig. 6). The distribution from *G. altivela* (Ateleopodiformes) had an antimode at around $2^8$ bp but, unusually, had a higher peak of long introns than short ones. With the exception of Zeiformes and Ateleopodiformes, distributions of the remaining species displayed an antimode position that is similar to *G. morhua* (Fig. 6).

## Discussion

### Bimodality of intron sizes

Bimodality is an interesting property as it suggests the presence of distinct classes that would be expected to share other properties. Our previous analyses of gene classes and intron sequences did suggest that long introns have evolved differently and are enriched in specific gene classes [12]; however, we did not observe a distinction around the distribution antimode but around $2^{10}$ to $2^{12}$ bp. Furthermore, ancestral state inference of intron size did not result in a distribution with a clear peak of very small introns as seen in almost all extant species. One interpretation of this is that there is no distinct set of short ( $2^8$ bp) introns and that the antimode exists not because of a natural distinction of intron classes but as a result of the mechanisms that underlie the evolution of intron size. Our observations here suggest that the loss of aKRAB from PRDM9 may have affected the evolution of intron sizes in teleosts leading to the TD.

### Two ways to lose aKRAB in teleosts

We identified PRDM9 candidate orthologues in 180 of the 185 species analysed; of these, we further analysed the sequences of proteins from 176 species. It seems likely that the failure to identify candidates from all species may be because of incomplete annotation as opposed to gene loss and that the PRDM9 content of the 176 species included in this analysis are largely representative of teleosts in general.

Our observations are consistent with a previous division of teleost PRDM9 orthologues into $\alpha$ (I) and $\beta$ (II, III, IV) types that arose as a result of the teleost specific genome duplication event [31]. We observed the $\beta$ type in all but one (a member of the Anguilliformes order, *G. javanicus*) of 176 species analysed; in contrast, less than half of the species contained the $\alpha$ type (Fig. 4). The lack of the aKRAB domain and an altered Zn finger array arrangement in all $\beta$ type orthologues indicate an early divergence of PRDM9-$\beta$ shortly after the teleost genome duplication event suggesting neo-functionalisation. The presence of PRDM9-$\beta$ in almost all teleosts analysed suggests that the new function is important but does not tell us what it is or whether it has any role in meiotic recombination.

Our observations indicate an early loss of the $\alpha$ type in the lineage leading to the Euacanthomorphacea (Percomorphs to Lampriformes) that contains more than half of all known teleost species. The loss of the $\alpha$ type in this lineage seems associated with an accelerated divergence of the PR-SET sequences in PRDM9-$\beta$ orthologues resulting in these sequences being more dis-similar to teleost $\alpha$ type sequences than these are to the corresponding mammalian sequences. In contrast, lineages outside of the Euacanthomorphacea typically contain both the $\alpha$ and $\beta$ types, and in these lineages, the $\beta$ sequence does not appear to have diverged as much. The divergence of the PR-SET domain sequence in the Euacanthomorphacea and the absence of the $\alpha$ type in this lineage suggests a modified molecular function; but again, we have no evidence as to what this might be.

Recent observations provide evidence for the role of salmonid PRDM9-$\alpha$ in directing meiotic recombination in a similar manner to that in mammals [47]. However, PRDM9-$\alpha$ proteins in about half of the teleost species analysed (26/45) did not appear to contain the aKRAB domain. As this is necessary for PRDM9 function in mammals, it seems likely that the ones lacking aKRAB have altered functions and that this has affected the meiotic recombination landscape.

The aKRAB domain is absent from most teleost species and orders. This loss seems likely to have resulted from a functional divergence of PRDM9-$\beta$ early in teleost evolution that included the loss of aKRAB followed either by the loss of PRDM9-$\alpha$, or losses of the aKRAB domain from PRDM9-$\alpha$ at later points. The repeated losses of the aKRAB domain within the teleosts suggest either the presence of alternative mechanisms for directing meiotic recombination or the loss of evolutionary selection for directed meiotic recombination.

### Loss of aKRAB and the rise of the TD

Evolution consists of two primary mechanisms: one, the generation of genetic variance, and two, the selection of the variance present within populations. Our findings indicate that the aKRAB domain has probably been lost independently eight times from teleost PRDM9 orthologues and that this loss has contributed to a distinct distribution of intron sizes. This necessitates that intron size has evolved differently in species with or without aKRAB; this could be either due to a change in generation of structural variance (the location and frequencies of insertion and deletions, indels) or to a change in the selection of structural variance.

The efficiency of selection across the genome depends on variation in local recombination rates [48, 49]; in areas of high recombination, selection is more efficient than in regions with low levels of recombination [50]. Hence, selection against the metabolic cost of long introns will be more efficient in regions of high recombination rates. Alternatively, the process of meiotic recombination itself contributes to the generation of structural variance as it involves both the induction and repair of DNA DSBs [51–54]. Consequently, differences in the spatial distribution of meiotic recombination events along the genome may have profound effects on the evolution of the genomic landscape.

Given the requirement of the aKRAB domain for PRDM9 function in meiotic recombination [36, 37], it seems unlikely that aKRAB-less orthologues play a role in determining locations of DSBs. The analysis of recombination patterns in swordtail fish (clade Percomorphaceae), which lacks an aKRAB domain, described an increase in recombination rates in regions associated with active transcription in the germline [31]. Similarly, Shanfelter et al. [55] observed an enrichment of recombination hotspots at transcription start sites of sticklebacks (*G. aculeatus*) but identified large differences in the locations of these hotspots between two populations which would be difficult to explain if germline gene activity was the only factor affecting meiotic site determination.

The simplest explanation of our observations is that the loss of aKRAB from PRDM9 orthologues in teleosts has altered the spatial distribution of recombination-related DSBs thereby affecting the spectrum of structural variance within introns. Ultimately, this may have contributed to the emergence of the TD over time. However, the absence of a TD in *S. formosus*, a few gadiform species, and the complete absence of PRDM9 in a number of other vertebrates (e.g. birds and canids) demonstrates that this is not sufficient in and of itself. The absence of the TD in *C. rupestris* may also be related to the time of the loss; we note that we observed aKRAB-like sequences in the *Bathygadus* genus which diverged from the *Coryphaenoides* about 50 million years ago (timetree.org [13]), whereas *D. clupeoides* diverged from other Clupeiformes about 150 million years ago, and it may be that it takes considerable time for the TD to evolve.

### Parallel evolution of non-adaptive traits

Although the size of introns does have a metabolic cost from the additional transcription required to express genes, it does not per-se mean that small introns will be selected for during evolution. This is because the additional cost of transcription for any given intron is likely to be extremely small compared to the total metabolic requirements of the cell [56]. This means that large introns can only be selected against in species that have extreme effective population sizes or for genes that are extremely highly expressed. The linear relationship between genome and intron sizes argues that introns in general evolve by mechanisms that are similar to intergenic regions and that the fact of being expressed has little effect on the evolution of intron size.

Nevertheless, here, we appear to observe the parallel emergence of a specific intron size distribution in multiple teleost clades. How can this happen in the absence of selection? The trait that we observe is bimodality with a specific antimode size. Intron size distributions are formed from a mixture of linear and exponential functions. The exponential nature comes from the fact that long introns are more likely to experience indel mutations that change their size; however, introns have a minimum size and mutations that would cause an intron to become smaller than this size will be strongly selected against (as they would in most cases lead to a non-functional protein). In the presence of a surplus of deletions leading to a general genome size contraction as appears to have happened in the teleosts [12], the proportion of introns of a minimal size will gradually accumulate. Long insertions of a fixed size (as would be the case for transposable elements) into the excess of minimal introns would then lead to a crossing of the antimode. Changing the relative probabilities of different types of insertion and deletion events within introns could thus lead to a bimodal intron size distribution in the absence of selection for intron size per se. This does not preclude selection for the causative event, and it is possible that the loss of the aKRAB domain has been selected for in the teleosts for other reasons.

## Conclusions

Teleosts have two types of PRDM9 orthologues; the predominant PRDM9-$\beta$ is present in almost all or all teleosts, whereas PRDM9-$\alpha$ is only found in non-Euacanthomorphacea lineages. The ancestral KRAB domain is absent from the $\beta$ type and has been lost independently from the $\alpha$ type in a number of lineages.

We show that the aKRAB domain is absent from all species with a TD but present in almost all lineages with non-TD distributions, arguing that the loss a aKRAB from PRDM9 is required for the TD to evolve. The appearance of the TD in multiple lineages is an example of convergent evolution of a trait (intron size distribution) which is likely to be non-adaptive. Our observations thus demonstrate how changes in molecular mechanisms can lead to the recurrent evolution of complex traits in the absence of selection for the trait itself and highlight the role meiotic recombination has in shaping genomes.

## Methods

All analyses were performed using a mixture of R, perl and shell commands found in scripts with suffixes of ‘.R,’ ‘.pl’ and ‘.sh’ respectively. Data visualisation was generated using core R functions, ggtree [57] and ggplot2 [58]. Detailed descriptions of methods is provided in the supplementary material.

### Teleost taxonomy and phylogeny

All taxonomic identification in this study is taken from the NCBI taxonomy. To consider the phylogenetic relationships among species included in this study, we made use of the TimeTree resource (http://timetree.org/ [13]; accessed 09/11/2022) to obtain phylogenetic trees.

### Distribution of teleost intron sizes

Genome annotations were retrieved as ‘.gff’ files from NCBI using the NCBI datasets utility. Intron coordinates from gff annotation files were extracted using a pair of shell scripts (Teleostei_IntronSizes_/GenBank/RefSeq.sh). These make use of exon coordinates and are adjusted for the difference in gff formatting between annotations retrieved from GenBank and RefSeq.

### PRDM9 structures

Gene, transcript and coding sequence coordinates were extracted from ‘.gff’ files obtained from NCBI. Genes whose annotation terms contained the term ‘PRDM9’ (case insensitive) were considered as candidate PRDM9 orthologues. The gene coordinates of candidates were used to extract and translate the candidate open reading using Biostrings version 2.64.1 [59]. The candidate teleost and mammalian (mouse, human) protein sequences were scanned by ‘interproscan’ [42] for matches to motifs and domains defined in the Pfam, SUPERFAMILY, Gene3D, ProSiteProfiles, PRINTS and Coils databases. We restricted further analyses to domains from ‘ProSiteProfiles’ since this was the only database with models matching the ancestral KRAB domain in teleosts. The coordinates of domains and introns were aligned by the last intron of the first (if multiple) PR-SET domain for visualisations.

The amino acid sequences of exons containing the PR-SET domain were extracted and aligned by the ‘msa’ package (version 1.28.0) [60] using the ‘msaClustalOmeage’ function with substitutionMatrix=‘BLOSUM65’ and “order=‘aligned’” options. A consensus matrix was derived from the alignments and a consensus score calculated for each sequence, and the alignment redone with sequences with scores below 250 removed. The resulting alignment was exported and a hidden Markov model (HMM) built with hmmbuild v3.3.2 [61] creating the ePR-SET HMM.

We obtained protein sequences from 213 annotated teleost genomes using the NCBI datasets utility. One hundred eighty-five of these coincided with those for which we had also obtained gene coordinates, and these were used for further analyses. We scanned all proteins for matches to the ePR-SET and aKRAB [44] HMMs using hmmsearch v.3.3.2 [61] as well as Prosite SET (PS50280), Krab related (PS50806), and C2H2 Zinc finger (PS50157) models using pfscanV3 v3.2.12 [42].

The intron and resulting domain coordinates were combined into a tabular format (parse_domain_scans.pl), and these were further analysed in R (R3/protein_strutures.R). To simplify the analysis, we identified the transcript with the largest number of distinct domains (or largest length of domain content for ties) as canonical and used these for further analyses.

Sequences matching the ePR-SET HMM with scores larger than 180 were extracted and aligned as above. Distances between aligned sequences were calculated as $\frac{\sum _i{D_{a[i],b[i]}}}{n}$ where *a* and *b* represent pairs of sequences, *i* the set of coordinates of residues aligned in the multiple sequence alignment for *a* and *b*, *n* is the size of the set *i* and *D* is a distance matrix derived from the Blosum62 matrix where $D_{x,y} = -B_{x,y} + (B_{x,x} + B_{y,y})/2$ where *x* and *y* represent a pair of residues and *B* is a Blosum substitution matrix (obtained from the source code of BLAST+ 2.9.0 [62]).

A second multiple sequence alignment and set of PR-SET distances was created that also included the mouse PRDM9 PR-SET sequence. These distances were used to create a rooted neighbour-joining tree using the bionj function of the ape package (version 5.7.1) [63]. This was used to define the order of proteins in Fig. 3A and C and is shown in Fig. 4.

### aKRAB genome scans

To scan 6-frame translated teleost genome assemblies for the presence of aKRAB with ‘hmmersearch’ (HMMER v3.3.2.; [61]), we made use of a PRDM9-specific aKRAB-A HMM profile obtained from Lorenz et al. [44].

### Confirmation of aKRAB loss from raw reads

To verify the absence of aKRAB in the order Zeiformes, in *G. altivela* (Ateleopodiformes) and several gadiform species, we downloaded raw sequencing data for each species from the NCBI SRA database. The blast databases of the raw sequencing reads were searched against the PRDM9 protein from *G. morhua* (Gadiformes; XP_030231029.1) using tblastn [62]. We restricted our search to the first 250 amino acids of the protein, which includes aKRAB and neighbouring sequences.

### Genome annotation

For each assembly of interest, repeat characterisation was performed using RepeatMasker v4.1.5 [64]. Braker3 v3.0.3 [65] was then run on the genome assemblies using the ‘vertebrate’ portion of the OrthoDB protein database as reference protein sequences. Intron sizes were determined using genomic coordinates from the gene annotation files generated by braker3.

## Supplementary information

Additional file 1: Figure S1 - Teleost intron size distributions.

Additional file 2: Supplementary methods and figures. More detailed descriptions of the methods used including the names of scripts where methods were implemented. Figures S1-S19: Figure S1 - Teleost intron size distributions. Figure S2 - Numbers of PRDM9 orthologue candidates. Figure S3 - Domain structure of PRDM9 orthologue candidates. Figure S4 - Domain structures of PRDM9 orthologue candidates. Figure S5 - Protein domain combinations. Figure S6 --SET and aKRAB scores. Figure S7 - Protein groups defined by two-SET domain models. Figure S8 - Mouse and human ePR-SET scores. Figure S9 - Avian ePR-SET scores. Figure S10 - Domain structure of PRDM9 orthologue candidates. Figure S11 - Numbers of PRDM9 orthologue candidates. Figure S12 - Domain structures of PRDM9 orthologue classes. Figure S13 - Domain sequence distance distributions. Figure S14 - aKRAB single best one domain score. Figure S15 - aKRAB losses in Osteoglossiformes. Figure S16 - aKRAB loss in Clupeiformes. Figure S17 - Coverage plots of tblastn search. Figure S18 - aKRAB distribution in Gadiformes. Figure S19 - Distinct distributions of teleost intron sizes. Scripts and data produced as part of the analysis can be found at https://doi.org/10.5281/zenodo.13918096..

## Data Availability

Data analysed in this work has been obtained from public repositories. Results of analyses performed, and the code implementing them, are provided as part of the supplementary materials and at 10.5281/zenodo.13918096.

## Abbreviations

TD: Teleost distribution
PRDM: PRDI-BF1 and RIZ homology domain containing
KRAB: Krüppel-associated box
aKRAB: Ancestral KRAB
KRAB-R: KRAB-related
SSXRD: SSX repression domain
PR-SET: PRDF1-RIZ-Su(var)3-9/Enhancer of Zeste/Trithorax
ePR-SET: Extended PR-SET
Zn: Zinc
C2H2: Cys2-His2
HMM: Hidden Markov model
C: Carboxyl
N: Amino
DSB: Double-strand break
