# Supplementary figures and images for: Multiple losses of aKRAB from PRDM9 coincide with a teleost-specific intron size distribution
Source: BMC Biol. 2024 Nov 27;22:275. doi: 10.1186/s12915-024-02059-w (PMC11600626; doi:10.1186/s12915-024-02059-w)

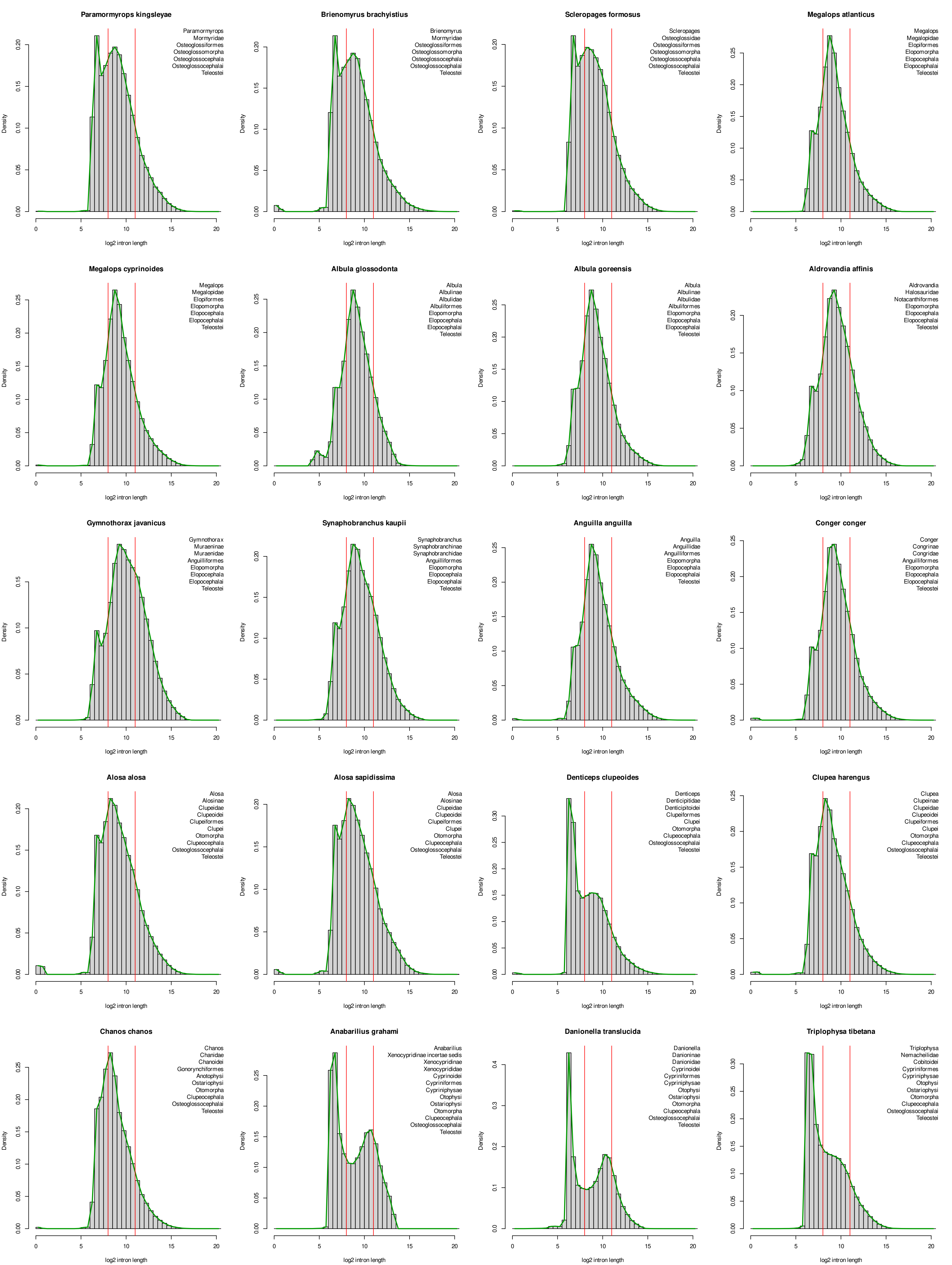

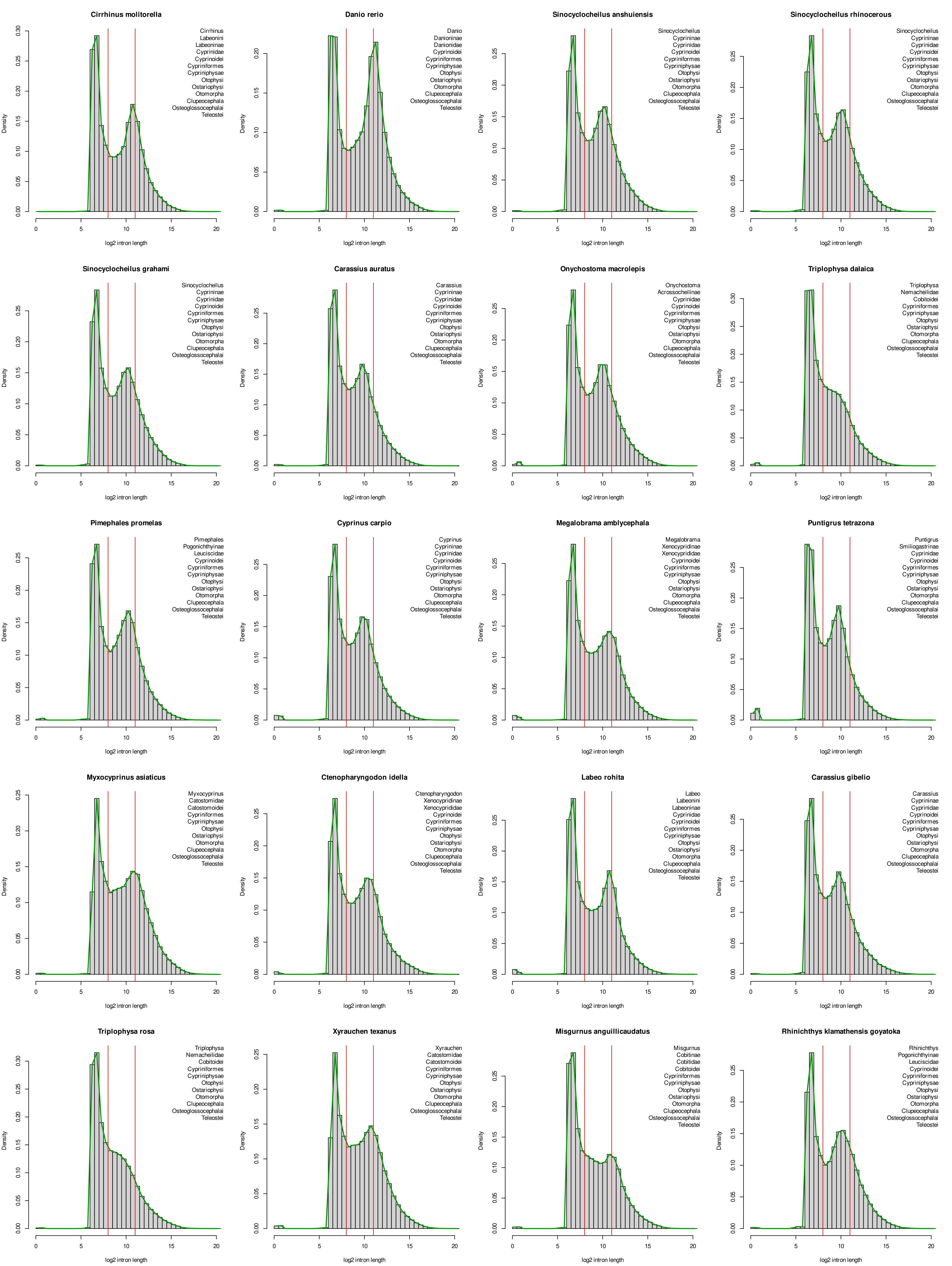

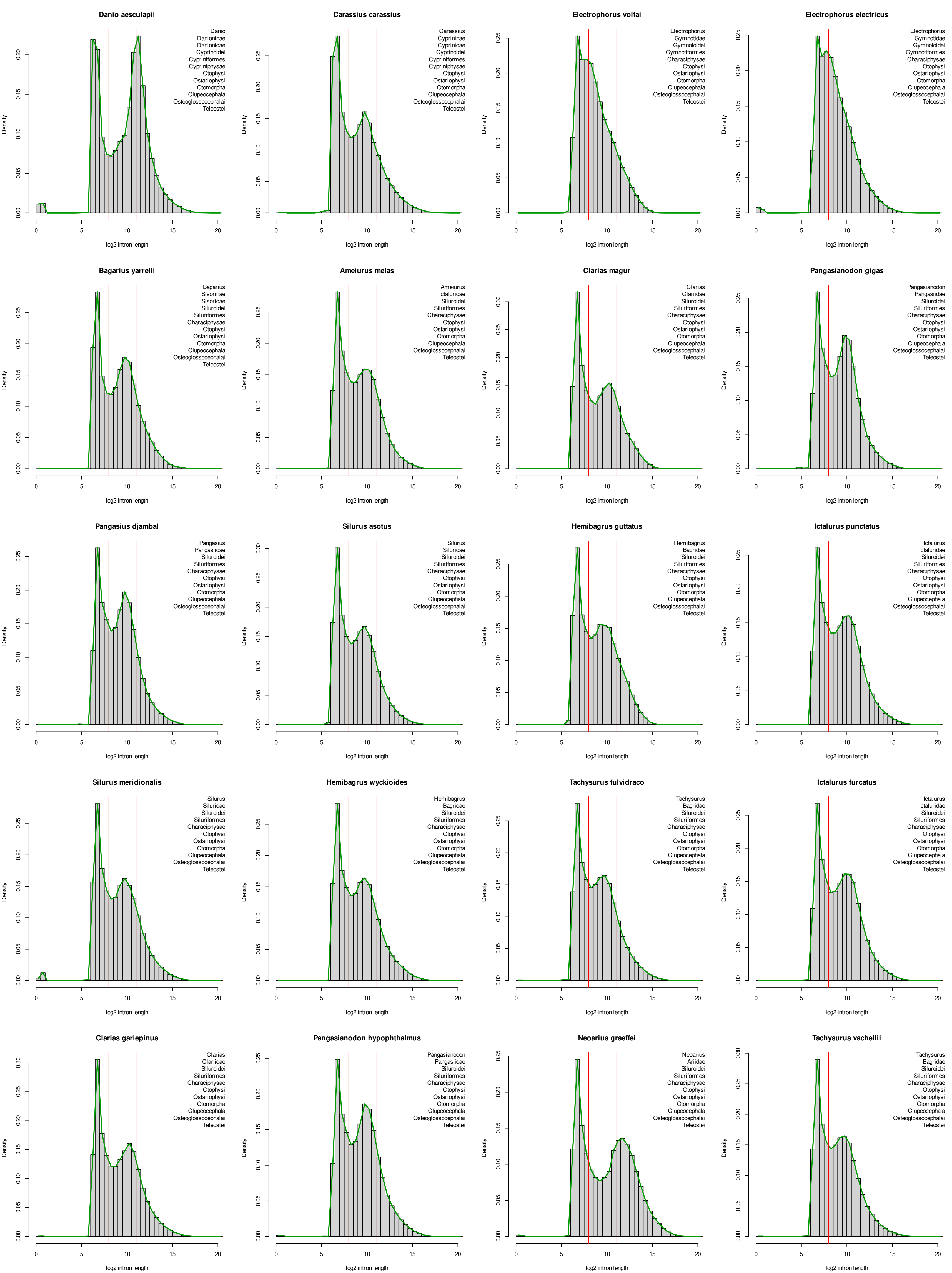

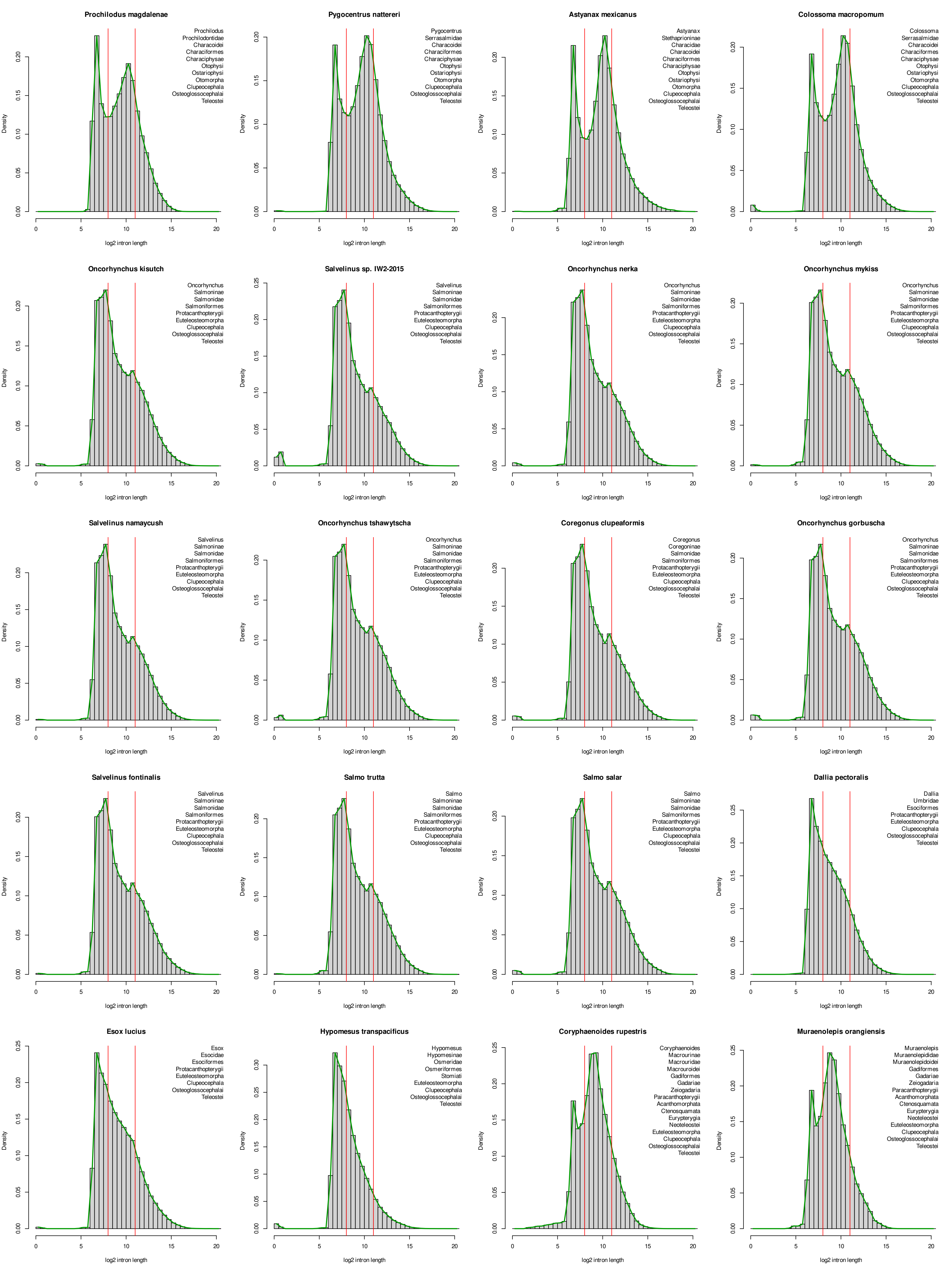

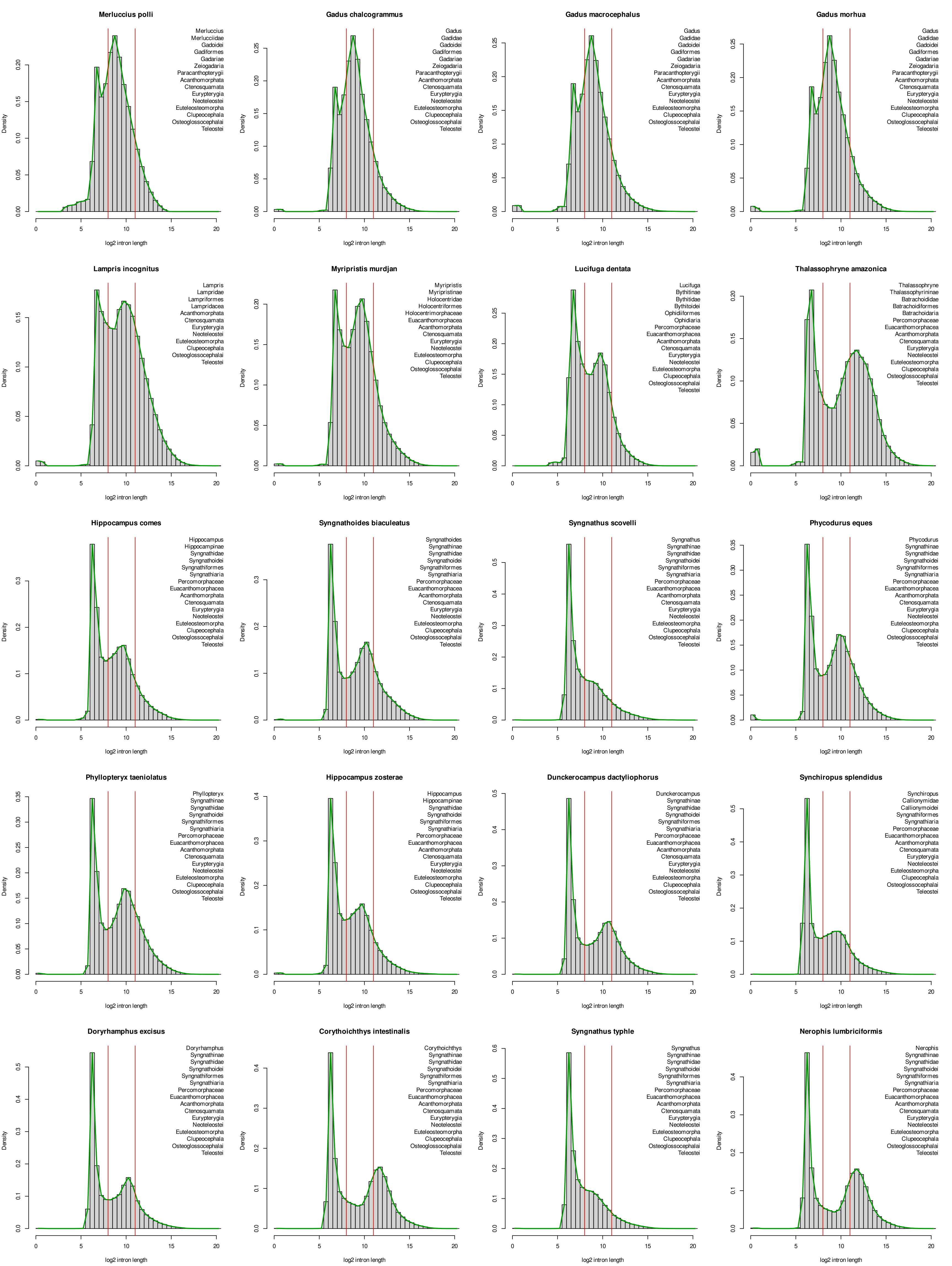

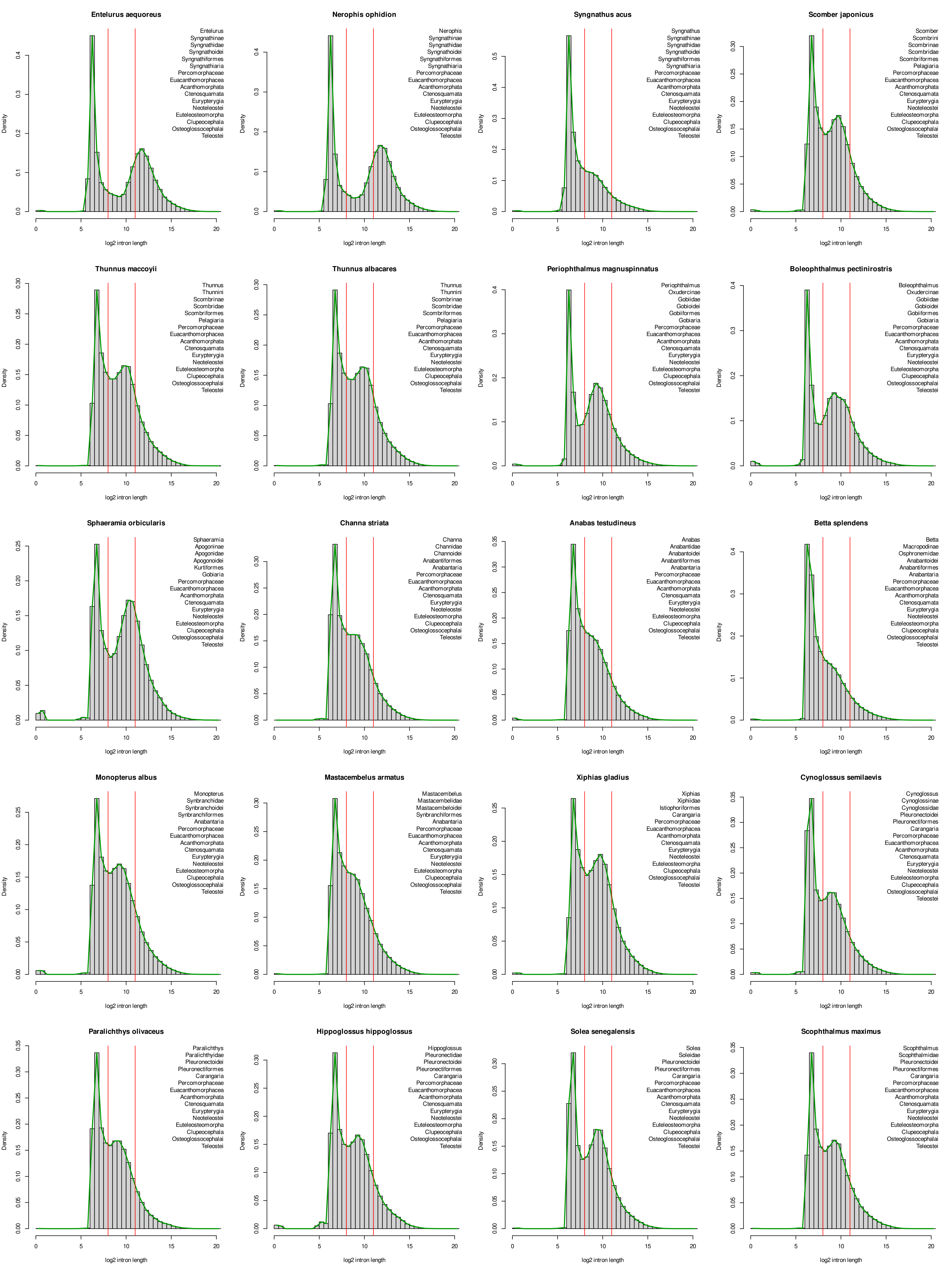

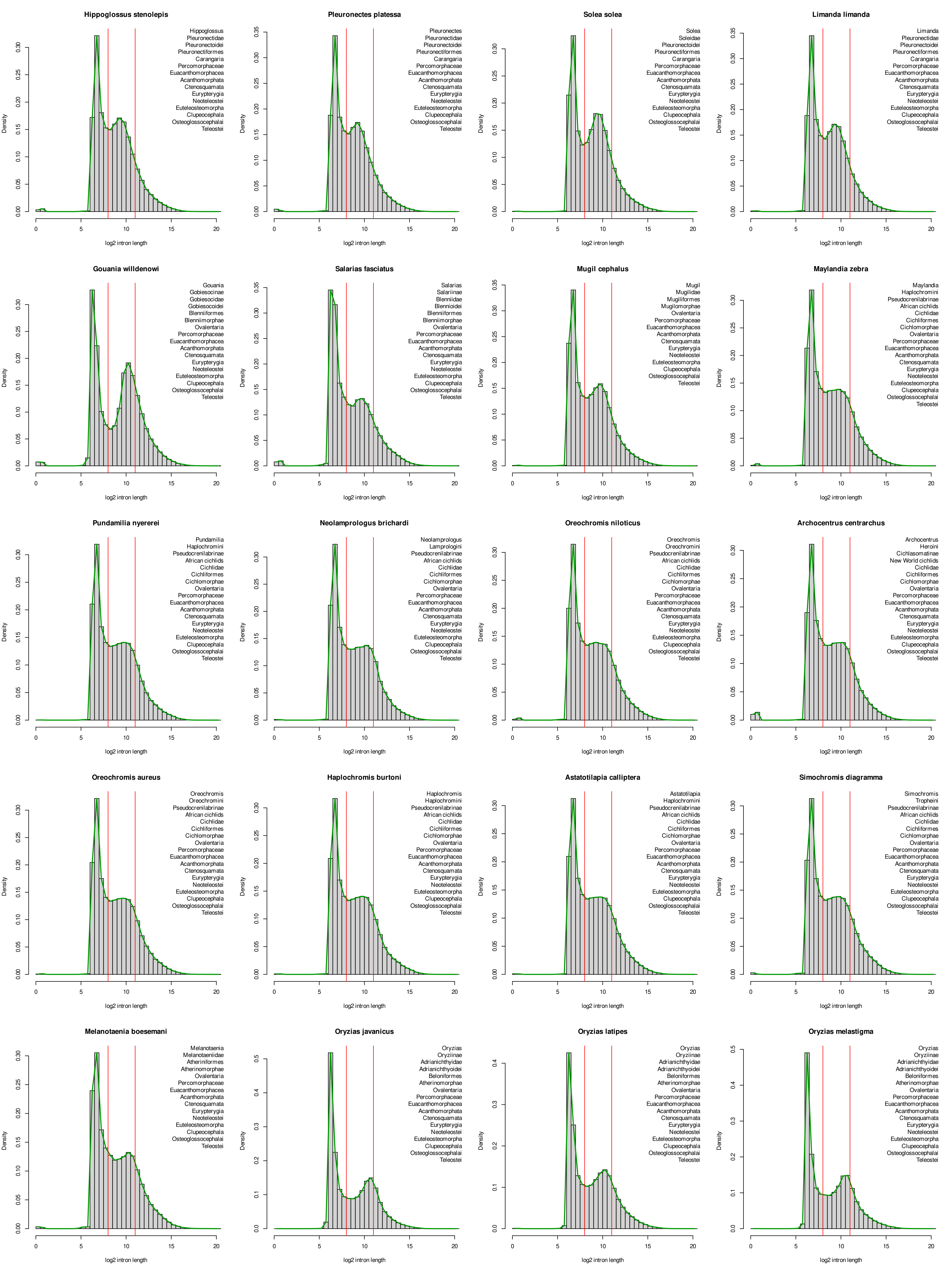

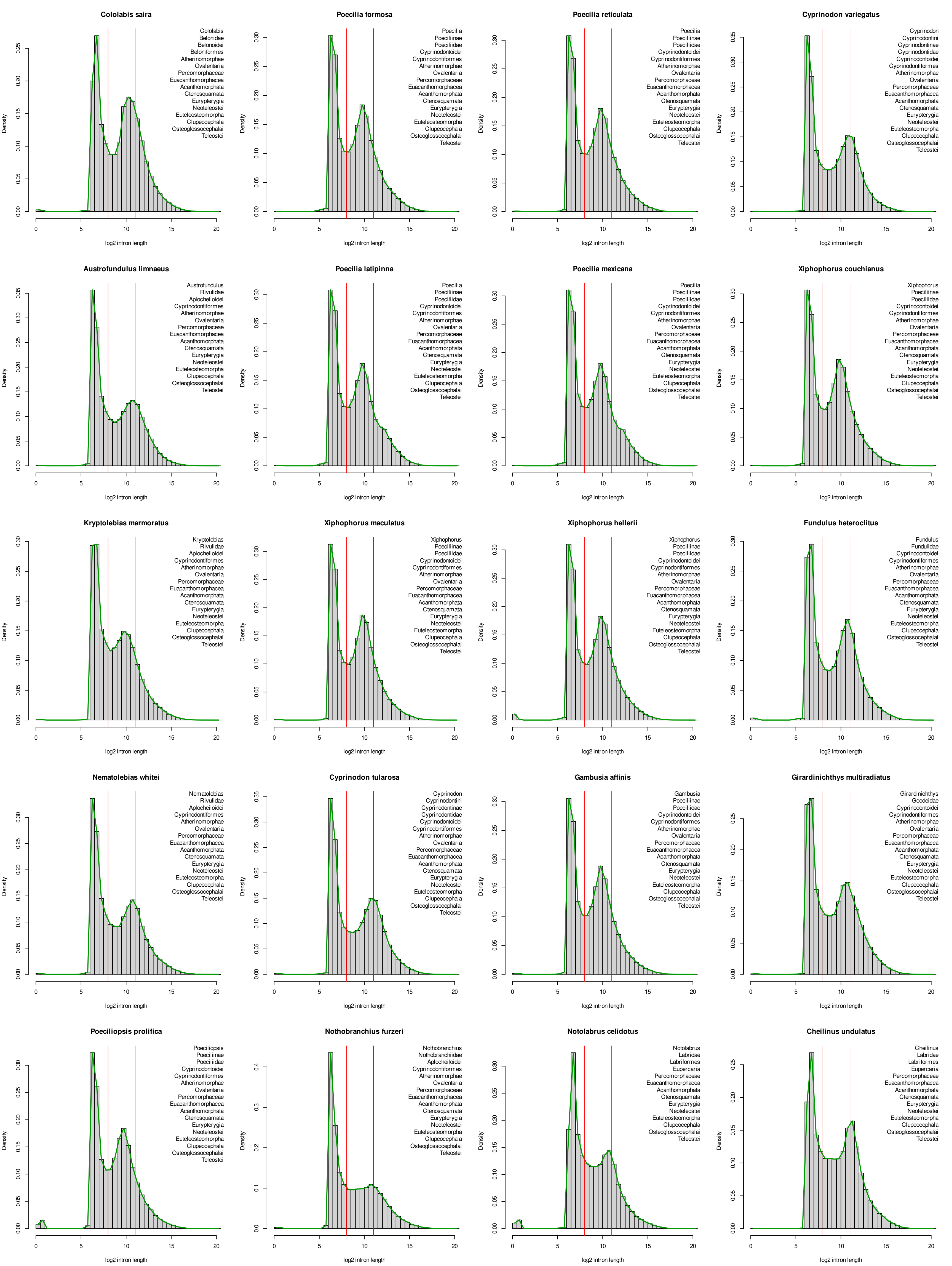

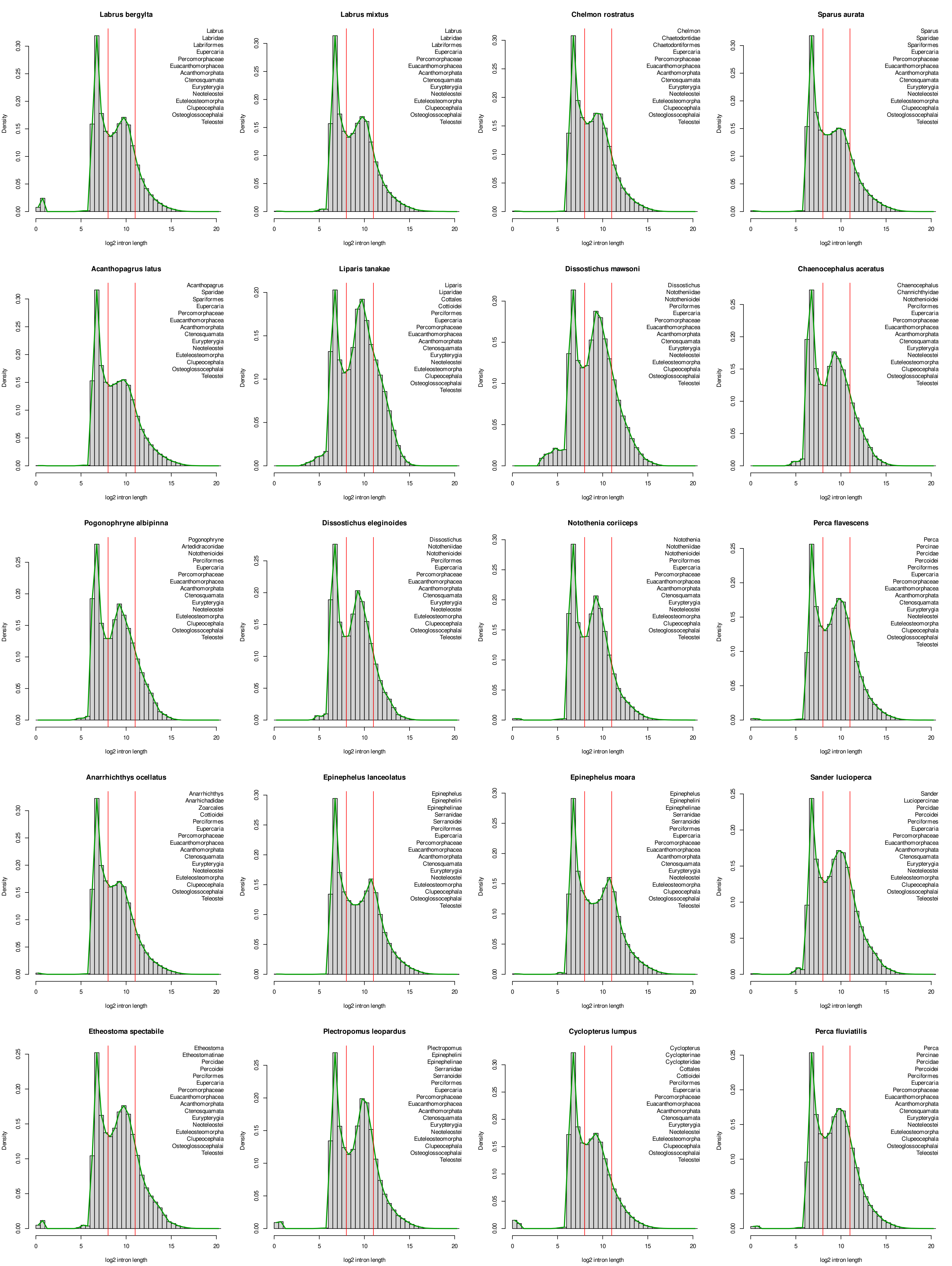

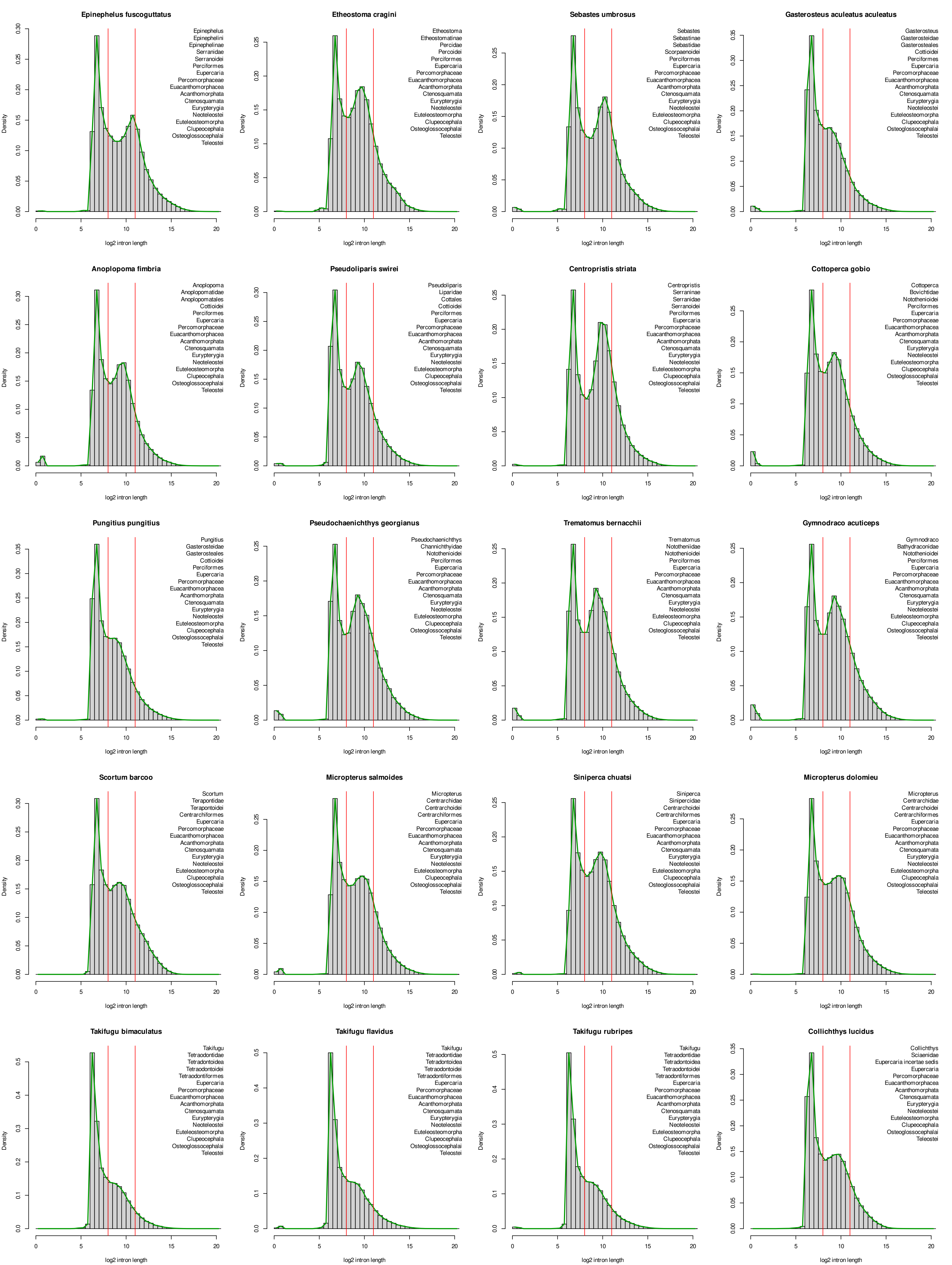

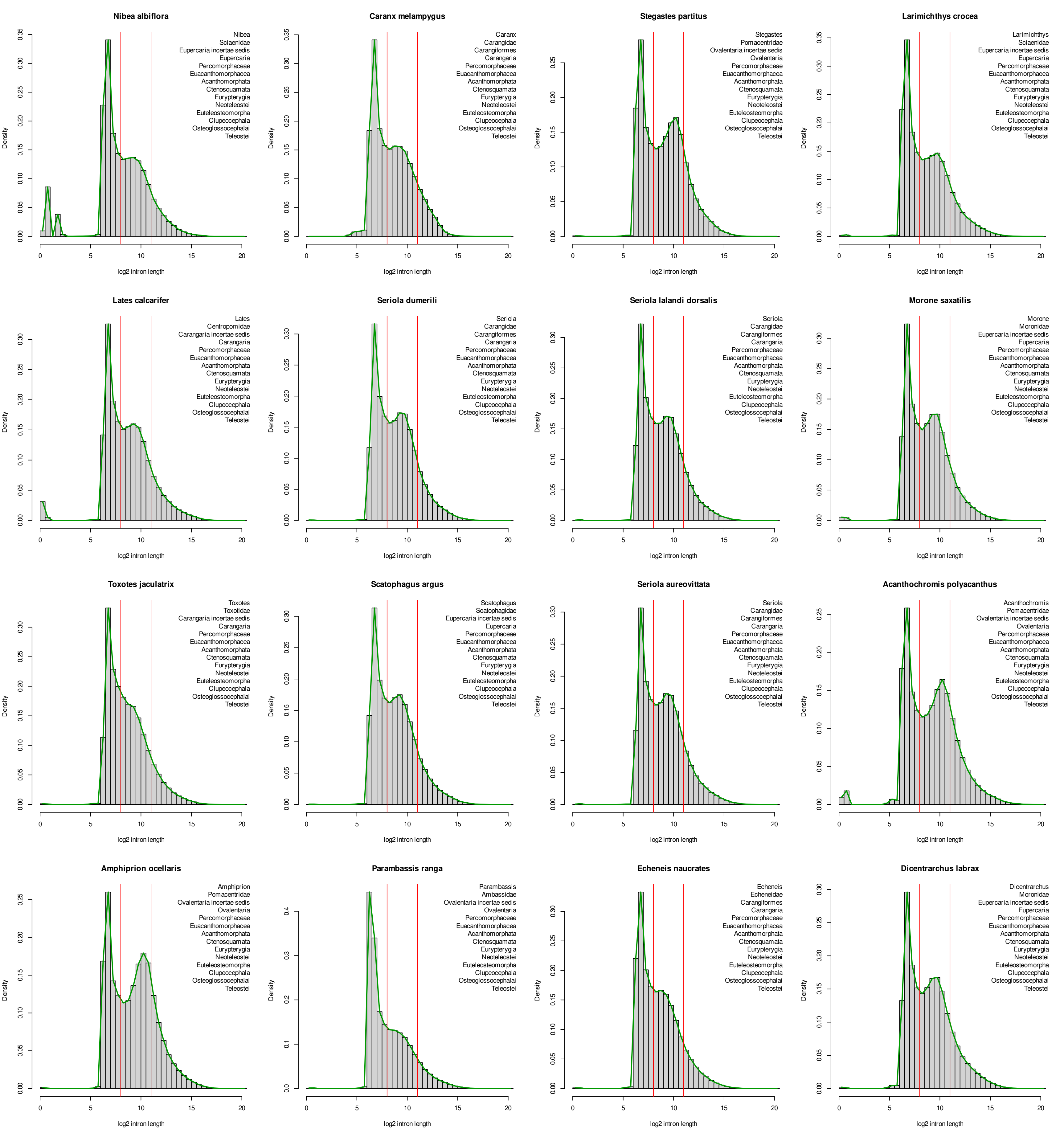

Supplement: Supplementary file 1 — Additional file 1: Figure S1 - Teleost intron size distributions. [file 12915_2024_2059_MOESM1_ESM.pdf]
