# Supplementary material for: Multiple losses of aKRAB from PRDM9 coincide with a teleost-specific intron size distribution
Source: BMC Biol. 2024 Nov 27;22:275. doi: 10.1186/s12915-024-02059-w (PMC11600626; doi:10.1186/s12915-024-02059-w)
Supplement: Supplementary file 2 — Additional file 2: Supplementary methods and figures. More detailed descriptions of the methods used including the names of scripts where methods were implemented. Figures S1-S19: Figure S1 - Teleost intron size distributions. Figure S2 - Numbers of PRDM9 orthologue candidates. Figure S3 - Domain structure of PRDM9 orthologue candidates. Figure S4 - Domain structures of PRDM9 orthologue candidates. Figure S5 - Protein domain combinations. Figure S6 --SET and aKRAB scores. Figure S7 - Protein groups defined by two-SET domain models. Figure S8 - Mouse and human ePR-SET scores. Figure S9 - Avian ePR-SET scores. Figure S10 - Domain structure of PRDM9 orthologue candidates. Figure S11 - Numbers of PRDM9 orthologue candidates. Figure S12 - Domain structures of PRDM9 orthologue classes. Figure S13 - Domain sequence distance distributions. Figure S14 - aKRAB single best one domain score. Figure S15 - aKRAB losses in Osteoglossiformes. Figure S16 - aKRAB loss in Clupeiformes. Figure S17 - Coverage plots of tblastn search. Figure S18 - aKRAB distribution in Gadiformes. Figure S19 - Distinct distributions of teleost intron sizes. Scripts and data produced as part of the analysis can be found at https://doi.org/10.5281/zenodo.13918096.. [file 12915_2024_2059_MOESM2_ESM.pdf]

Supplementary material for:

Multiple losses of aKRAB from PRDM9 coincide with a teleost-specific intron size distribution

Ann-Christin Zinner and Lars Martin Jakt

Contents

General overview 3

Teleost phylogeny and taxonomy 5

Genome assemblies, annotation and protein sequences 5

Vertebrate (non-teleost) genome annotation 5
Teleost genome annotation 5
Genome assemblies 5
Protein sequences 6

Intron size distributions 6

Non-teleost vertebrates 6
Teleosts 7
Classification of intron size distributions 7

Teleost PRDM9 domain structures 7

Hmmer scan for aKRAB 8

Identifying KRAB motifs from raw reads with tblastn 9

Genome annotation 9

Data files 10

Tables 10

Scripts **10**
Data retrieval and taxonomy 10
Domain structure analyses 11
Intron sizes 13
aKRAB in genomic sequences 14

Supplementary Figures **15**

General overview

The analyses can be divided into the following steps:

- Retrieval of teleost genome annotation and extraction of intron coordinates from gff files to obtain teleost intron size distributions.
- Analysis of PRDM9 gene structure in teleosts using existing annotation and protein sequences.
- Retrieval of teleost genome assemblies and 6-frame translations of all assemblies for hmmer search for the aKRAB domain of PRDM9.
- Genome annotation of assemblies for species of interest.

To aid the reader we have used a monospaced font to denote the names of files, directories and programs throughout this text. Hence, `R3/dom_int_can_set_pos.tsv` indicates the name of a file present in the subdirectory `R3`. Similarly, `hmmsearch` indicates the name of a program used as part of the analysis.

Almost all programs used were run from shell scripts provided as part of the supplementary materials in order to preserve the options used, and to indicate the names of input and output files. A small number of perl scripts were run with file names hard coded into the script. The names of the scripts used to run the analyses are given in brackets after the description if not stated explicitly. e.g. Intron coordinates were extracted from gff files (`extract_coords.sh`), indicating that the commands used to extract coordinates can be found in the `extract_coords.sh`.

Most of the final analyses and data visualization was carried out in R. A number of shell and perl scripts were used to run programs (eg. `hmmsearch`) and to extract data into formats suitable for analysis in R. Note that the analyses were not necessarily performed in the order listed above. R, shell and perl scripts are denoted by their respective suffixes (`.R`, `.sh`, and `.pl`) throughout the rest of this document.

Scripts and code used for analyses and data processing can be found in `supplementary methods/code`; the data used in this study is accessible in `supplementary methods/data_supp` which has several sub directories. All figures with associated code to generate these figures are located in `'supplementary methods/figures'`; they were created using core R functions and ggtree [1] with ggplot2 [2]. `supplementary methods/teleost_trees` contains the newick files of the pylogenetic trees used in this study.

All figures were created using R; the names of the image files (usually in `.pdf` format) containing the figures are provided in table 1. The exact methods used to construct the plots can be identified by searching the `R` source files for the name of the image file and then working backwards to determine how the data objects used were created and the source of data used in the analyses. The source code provided is the authoritative description of what was done; in addition we provide a an explanation of the procedures followed and the relationships between different source and data files.

Table 1 Figures and their sources: Column one gives the figure number, with S indicating supplementary figures. The 'Data' column indicates data processed by the 'Script' to provide the figure. In Some cases, too many input data files were used and in this case the 'Data' columns has been left empty.

Figure	Data	Script	Figure file
1		vertebrate_intron_distributions.R	vertebrate_intron_distributions.pdf
2	SI_Table_IntronSizes.tab, Teleostei_order.nwk	intron_sizes_teleost_orders.R	intron_sizes_teleost_orders.pdf
3		R3.2/protein_structure.R	prdm9_classes_figure_mr.pdf
4		R3.2/protein_structure.R	SET_mouse_rooted_tree.pdf
5	SI_Table_assemblies.tab, SI_Table_aKRAB_hmmer.tab, Teleostei_order.nwk	aKRAB_hmm_scores_tree.R	aKRAB_hmm_scores_tree.pdf
6	SI_Table_IntronSizes.tab, SI_Table_IntronSizes_braker.tab, SI_Table_aKRAB_hmmer.tab, Teleostei_order.nwk	distinct_dist.R	distinct_dist.pdf
S1	SI_Table_IntronSizes.tab, functions.R, teleost_tree.rds	teleost_intron_size_distribution.R	teleost_intron_size_distribution.pdf
S2		R1/protein_structure.R	R1/candidate_species_counts.pdf
S3		R1/protein_structure.R	R1/prdm9_dom_introns_summary_set.pdf
S4		R1/protein_structure.R	prdm9_dom_introns_summary_set.pdf
S5		R3.2/protein_structure.R	domain_combinations_canonical_th.pdf
S6		R3.2/protein_structure.R	krab_set_scores.pdf
S7		R3.2/protein_structure.R	SET_domain_model_scores_02.pdf
S8		R3.2/protein_structure.R	mammalian_prset_hmm_scores.pdf
S9		R3.2/protein_structure.R	avian_prset_hmm_scores.pdf
S10		R3.2/protein_structure.R	R3.2/SET_domain_summary_01.pdf
S11		R3.2/protein_structure.R	species_orthologue_counts_3.pdf
S12		R3.2/protein_structure.R	SET_domain_summary_al_msa_mr_o_01.pdf
S13		R3.2/protein_structure.R	prdm9_distances_distributions_mr.pdf
S14	SI_Table_aKRAB_hmmer.tab	aKRAB_hmm_scores.R	aKRAB_hmm_scores.pdf
S15	SI_Table_aKRAB_hmmer.tab, SI_Table_assemblies.tab, Osteoglossi-formes_genus.nwk	aKRAB_osteoglossiformes_genera.R	aKRAB_osteoglossiformes_genera.pdf
S16	SI_Table_aKRAB_hmmer.tab, SI_Table_assemblies.tab, Clupeiformes_genus.nwk	aKRAB_clupeiformes_genera.R	aKRAB_clupeiformes_genera.pdf
S17	*tblastn.out	tblastn_coverage.R	tblastn_coverage.pdf
S18	SI_Table_aKRAB_hmmer.tab, SI_Table_assemblies.tab, Gadiformes_genus.nwk	aKRAB_gadiformes_genera.R	aKRAB_gadiformes_genera.pdf
S19	SI_Table_IntronSizes.tab	manual_annotation.R	distribution_distinction.pdf

Teleost phylogeny and taxonomy

Taxonomic information was extracted from the NCBI taxonomy database (https://ftp.ncbi.nlm.nih.gov/pub/taxonomy/new_taxdump/new_taxdump.tar.gz) using `NCBItax2lin` v2.3.1 (https://github.com/zyxue/ncbitax2lin) called from `download_ncbi_tax.sh` (script 1). All species not belonging to the infraclass 'Teleostei' were removed from the resulting table (`tax_subset_teleostei.sh`, script 2) generating the table `SI_Table_Teleostei_taxonomy.tab` (data file 10).

Phylogenetic trees were obtained from the TimeTree database (http://timetree.org) [3]. Note that since the last accession (09/11/2022) minor changes to the phylogeny of the teleost orders have been made; mainly due to uncertainty in the placement of several orders of percomorphs. For this study, however, these are irrelevant, as no percomorphs have an aKRAB containing PRDM9 orthologue.

Genome assemblies, annotation and protein sequences

Genome assemblies, genome annotation, protein sequences and assembly metadata were obtained from NCBI using the NCBI Datasets command line tools (version: 14.6.5) called from shell scripts (see below for details).

Vertebrate (non-teleost) genome annotation

Scripts 3 to 7 were used to download genome annotation and assembly metadata for lobe-finned fishes (`download_sarcopterygii.sh` script 3), non-teleost ray-finned fishes (`download_chondrostei.sh`, script 4; `download_holostei.sh`, script 5; `download_cladistia.sh`, script 6) and cartilaginous fishes (`download_chondrichthyes.sh`, script 7). Species names and accessions are given in `info_chondrichthyes_annotations.tab` (data file 5), `info_chondrostei_annotations.tab` (data file 6), `info_cladistia_annotations.tab` (data file 7), `info_holostei_annotations.tab` (data file 8) and `info_sarcopterygii_annotations.tab` (data file 9).

Teleost genome annotation

Genome annotation and assembly metadata were downloaded using `download_teleostei_annotations.sh` (script 8). The shell script called the `datasets` command line to download genome annotation and `dataformat` command line tool to extract metadata (`info_teleostei_annotations_ncbi_02.tab`, data file 4).

Genome assemblies

Genome assemblies used in this study were obtained using a series of scripts calling the NCBI `datasets` and `dataformat` utilities to download and extract metadata respectively. Initially

download_teleostei_assemblies_ncbi_01.sh (script 9), and then for assemblies submitted after this, download_teleostei_assemblies_ncbi_02.sh on 20/05/2023 (script 10), and finally four chromosome-level assemblies were manually selected and downloaded (download_teleostei_assemblies_ncbi_03.sh, script 11). Assembly metadata is given in the info_teleostei_assemblies_ncbi_[0|1|2|3].tab files (??).

Assembly metadata files from each download were subsequently merged and only the newest assembly version for each species was used for further analyses. This resulted in assemblies for 1202 individual species. Each species was associated with its higher taxonomic levels (genus, family, order) by mapping species names to the NCBI taxonomy (info_assemblies.R, script 13) giving SI_Table_assemblies.tab (data file 1). Nine assemblies did not download correctly. As these species were from orders that were well represented with the remaining assemblies, they were excluded from further analyses.

Protein sequences

Reference protein sequences of teleosts were downloaded using download_teleostei_protein_ncbi.sh (script 12) which called the NCBI command line utilities datasets and dataformat to download and extract metadata respectively. The set of assemblies used for these analyses is given R3.2/teleost_prot_assembly_info.tsv (data file 15).

Intron size distributions

Intron coordinates from gff annotation files were extracted using a pair of shell scripts (Teleostei_IntronSizes_GenBank.sh and Teleostei_IntronSizes_RefSeq.sh, scripts 1 and 2). These make use of exon coordinates and either the locus_tag (Genbank) or the gene (RefSeq) identifers to group exons by gene. The script outputs one bed file per species containing the sequence id (usually scaffold), intron start and end coordinates, gene id, strand and intron length for each intron. Only unique intron coordinates were output in order to avoid over-counting introns from splice variants. For convenience, these files were renamed to incorporate accession ids (label_by_accession.sh, script 7).

Non-teleost vertebrates

Intron sizes of non-teleost vertebrates, were determined from RefSeq annotation (gff) only. vertebrate_intron_distributions.R (script 3) was used to combine intron sizes with taxonomic information from info_chondrichthyes_annotations.tab (data file 5), info_chondrostei_annotations.tab (data file 6), info_cladistia_annotations.tab (data file 7), info_holostei_annotations.tab (data file 8), info_sarcopterygii_annotations.tab (data file 9) and vertebrate_taxonomy.tab (taxon clades, data file 11).

Teleosts

intron_size_ncbi.R (script 4) was used to combine intron sizes from all species with taxonomic information from info_teleostei_annotations_ncbi_02.tab (accession to species, data file 4) and SI_Table_Teleostei_taxonomy.tab (taxon classes, data file 10) giving SI_Table_IntronSizes.tab (data file 2). Of the downloaded gff annotation, we did not determine intron coordinates from five (GCA_000180735.1, GCA_033238685.1, GCA_902810595.1, GCA_907169785.1, GCA_962446985.1) as they either lacked information about exon coordinates or the formatting differed from the other annotation.

Classification of intron size distributions

Intron size distributions were classified three times manually in random orders as either TD (teleost specific distribution) non-TD or uncertain using an interactive R script. Distributions that had non-unanimous classifications (2) were considered as uncertain. Plots of the distribution density around the antimode against the density at longer lengths were then used to explore the potential for a simple classifier (Fig. S19) that was used to evaluate distributions from unannotated assemblies. The code used for this is found in intron_distributions_manual_classification/manual_annotation.R (script 8).

Teleost PRDM9 domain structures

Annotation for 191 assemblies in the gff format was obtained from NCBI as described above. Gene, transcript and coding sequence coordinates were extracted into a tabular format (feature files) using a perl script (extract_genes_exons_from_gff.pl, script 4). Candidate PRDM9 orthologues were identified from these using identify_gene_candidates.pl (script 6) that extracted genes containing the term "PRDM9" (case insensitive) in any annotation field. The feature files and the identified candidates were used by an R script (R1/protein_structure.R, script 20) that used the Biostrings package [4] to extract and translate the candidate coding sequences of PRDM9 orthologues. These sequences were written to a fasta file (R1/teleost_prdm9_pep.faa data file 13) that was scanned by interproscan [5] (R1/tel_interpro_scans.sh, script 11) for matches to motifs and domains defined in the Pfam, SUPERFAMILY, Gene3D, ProSiteProfiles, PRINTS and Coils databases. We followed a similar procedure to scan human and mouse PRDM9 orthologues (R1/nt_interpro_scans.sh, script 12). We restricted further analyses to domains from "ProSiteProfiles" since this was the only database with models matching the ancestral KRAB domain in teleosts. The interproscan output ([non_]teleost_prdm9_pep_<db>_1.tsv, data file 14) was loaded into the R session and the coordinates of domains and introns were aligned by the last intron of the first (if multiple) for SET domain visualisations.

The sequences of the exons containing the SET (PS50280) domain were extracted and aligned by the msa package [6] using the msaClustalOmeage function with substitutionMatrix='BLOSUM65', and order='aligned' options. A consensus

matrix was obtained for the alignments and a consensus score calculated for each sequence ($\sum S_{i,a[i]}$, where S is the consensus matrix, $a[i]$ denotes the residue at the ith position in each sequence), and the alignment redone with sequences with scores below 250 removed. The resulting alignment was exported and a hidden Markov model (HMM) built with `hmmbuild` version 3.3.2 (http://hmmer.org, `hmmer_profiles/build_hmm_PRSET_teleostei.sh`, script 24) creating the ePR-SET HMM.

We obtained protein sequences from 213 annotated teleost genomes using the NCBI `datasets` utility (`download_teleostei_protein_ncbi.sh`, script 12). 185 of these coincided with those for which we had also obtained gene coordinates and these were used for further analyses (data file 15). We scanned all proteins for matches to the ePR-SET and aKRAB [7] HMMs using hmmsearch (`run_hmmer_aKRAB-A.sh, run_hmmer_ePRSET_2.sh`, scripts 14 and 15) as well as Prosite SET (PS50280), Krab related (PS50806), and C2H2 Zinc finger (PS50157) models (obtained from https://github.com/sib-swiss/pftools3 using pfscanV3 (`run_prosite_PRDM9.sh`, script 16).

The intron and resulting domain coordinates were combined into a tabular format (`parse_domain_scans.pl`, script 17) and these were further analysed in R (`R3.2/protein_strutures.R`, script 22). To simplify the analysis we identified the transcript with the largest number of distinct domains (or largest length of domain content for ties) as canonical and used these for further analyses.

The sequences matching the ePR-SET HMM with scores larger than 180 were extracted and combined (`extract_hmm_set.pl`, script 19) on exported coordinates (`R3.2/dom_int_can_set_pos.tsv` data file 16). The sequences were aligned as above and distances between aligned sequences calculated as $\frac{\sum_i D_{a[i],b[i]}}{n}$ where a and b represent pairs of sequences, i the set of coordinates of residues aligned in the multiple sequence alignment for a and b, n is the size of the set i and D is a distance matrix derived from the Blosum62 matrix where $D_{x,y} = -B_{x,y} + (B_{x,x} + B_{y,y})/2$ where x and y represent a pair of residues and B is a Blosum substitution matrix (obtained from the source code of the Blast [8] application). The resulting SET distances were visualised as a heatmap and four distinct classes were inferred by visual inspection.

A second multiple sequence alignment and set of SET distances was created that also included the mouse PRDM9 SET sequence was constructed as above. These distances were used to create a rooted neighbor-joining tree using the `bionj` function of the ape package [9]. This was used to define the order of proteins in in figure 2 A and C and is shown in figure 3.

Hmmer scan for aKRAB

The search of teleost assemblies for the aKRAB domain was carried out with a shell script (`hmmer_aKRAB.sh`, script 2). The script calls a custom perl script (`extract_orfs.pl`, script 1) that 6-frame translates each assembly; translated open reading frames were then piped to the `hmmsearch` function of the HMMER suite [10].

We used an aKRAB-A specific profile from Lorenz et al. [7], with an evalue threshold of 1. The shell script saves the result in multiple formats: in addition to the standard output format (see [10]), hits are also saved in a tabular output file (`--tblout`) and as an alignment (`-A`). All hits were then summarized with an R script (`parse_hmmer.R`, script 3) that reads in all results in tabular format using the `read_tblout` function of the rhmmer package. It makes use of the `SI_Table_assemblies.tab` (data file 1) to match assembly accessions to species. It outputs a summary table of the results from the hmmer scan for aKRAB for all assemblies analyzed (`SI_Table_aKRAB_hmmer.tab`, data file 3).

Identifying KRAB motifs from raw reads with tblastn

Raw sequencing data was downloaded for two species of the order Zeiformes (*Cyttopsis rosea, Zeus faber*), *Guentherus altivela* (Ateleopodiformes) and multiple species of gadiform fish (*Arctogadus glacialis*, *Bregmaceros cantori* , *Brosme brosme*, *Coryphaenoides rupestris*, *Gadiculus argenteus*, *Macrourus berglax*, *Malacocephalus occidentalis*, *Melanogrammus aeglefinus*, *Melanonus zugmayeri*). Sequencing runs with the largest number reads for each species were identified manually (`tblastn_species_accession.tab`, data file 17) and the sequence data were obtained using NCBI's SRA Toolkit's `prefetch, fastq-dump, fasterq-dump` called from (`download_sra.sh`, script 4). Blast [8] databases were built with 'makeblastdb' (`make_blastdb.sh`, script 7), that is part of BLAST command line applications. `tblastn` was called from `run_tblastn.sh` (script 8) with the *G. morhua* PRDM9 protein (XP_030231029.1) as a query, restricting the search to the first 250 amino acids with `-query_loc`, keeping a maximum of 2000 aligned sequences (`-max_target_seqs`) with an evalue threshold of 0.05 (-evalue). Genome assemblies were indexed with bwa (`bwa index [assembly]`) and each set of raw sequences were aligned to their respective genome assemblies using bwa mem with standard settings and piped to samtools [11] (`bwa mem -t 8 [assembly] [_1.fastq] [_2.fastq] | samtools sort -o _sorted.bam`). Read depth at each position was determined with samtools depth (`cov_expected.sh`, script 6).

Genome annotation

We estimated intron sizes for a number of unannotated assemblies. Repeat characterization for each assembly was performed with RepeatMasker v4.1.5 [12] with the species parameter set to 'Teleostei' and hmmer as the search engine. The repeat-masked genome was then annotated with Braker3 v3.0.3 [13] using the 'vertebrate' portion of the OrthoDB protein database as reference protein sequences (`--prot_seq`). Due to the fragmented nature of the assemblies, the minimal contig length was set to 5000 and parallelization was skipped (`--threads=1 --min_contig=5000`). Intron sizes were determined using genomic coordinates from the resulting gtf files.

Data files

Tables

1. SI_Table_assemblies.tab Assembly information (accession, level, contigN50, scaffoldN50, submission date) and species information (name, order, family, clade).
2. SI_Table_IntronSizes.tab
   Intron sizes for 216 assemblies with species information (name, order, clade).
3. SI_Table_aKRAB_hmmer.tab
   Hmmer results of 1193 assemblies with species information (name, family, order, clade).
4. info_teleostei_annotations_ncbi_02.tab
   Assembly accession and organism name of annotated genomes.
5. info_chondrichthyes_annotations.tab
   Assembly accession and organism name of annotated genomes.
6. info_chondrostei_annotations.tab
   Assembly accession and organism name of annotated genomes.
7. info_cladistia_annotations.tab
   Assembly accession and organism name of annotated genomes.
8. info_holostei_annotations.tab
   Assembly accession and organism name of annotated genomes.
9. info_sarcopterygii_annotations.tab
   Assembly accession and organism name of annotated genomes.
10. SI_Table_Teleostei_taxonomy.tab
    Teleostei taxonomy information.
11. vertebrate_taxonomy.tab
    Vertebrate taxonomy information.
12. SI_Table_IntronSizes_braker.tab
    Intron sizes for eight species annotated in this study.
13. R1/teleost_prdm9_pep.faa
    Sequences of candidate teleost PRDM9 orthologues that contained the term "PRDM9" in their annotation.
14. R1/[non_]teleost_prdm9_pep_<db>_1.tsv
    A series of files containing the results of Interproscan scans against non-teleost PRDM9 orthologues (mouse and human) and teleost candidate orthologues. <db> indicates the name of the database for each file. These files are provided as a single zipped archive, R1/prdm9_pep_interproscan.zip.
15. R3.2/teleost_prot_assembly_info.tsv
    Assembly metadata for the set of teleost assemblies from which protein sequences were scanned for various domain models.
16. R3.2/dom_int_can_set_pos.tsv
    Coordinates of extended PR-SET (ePR-SET) domains in teleost proteins.
17. tblastn_species_accession.tab
    Accession codes and species identifiers for raw reads used to confirm absence of aKRAB from selected assemblies.

Scripts

Data retrieval and taxonomy

1. download_ncbi_tax.sh
   Used to download the NCBI taxonomy database taxdump and extract relevant data to a tabular format by calling ncbitax2lin; outputs comma separated file, ncbi_lineages_2024-01-25.csv.
2. tax_subset_teleostei.sh
   Extracted the 'Teleostei' subset of the NCBI taxonomy from file generated by download_ncbi_tax.sh. Generated SI_Table_Teleostei_taxonomy.tab.
3. download_sarcopterygii.sh
   Downloads all Sarcopterygii annotation with reference status in gff format to sarcopterygii_annotations and generates the file info_sarcopterygii_annotations.tab containing accession and species name.

4. `download_chondrostei.sh`
Downloads all Chondrostei annotation with reference status in `gff` format to `chondrostei_annotations` and generates the file `info_chondrostei_annotations.tab` containing accession and species name.
5. `download_holostei.sh`
Downloads all Holostei annotation with reference status in `gff` format to `holostei_annotations` and generates the file `info_holostei_annotations.tab` containing accession and species name.
6. `download_cladistia.sh`
Downloads all Cladistia annotation with reference status in `gff` format to `cladistia_annotations` and generates the file `info_cladistia_annotations.tab` containing accession and species name.
7. `download_chondrichthyes.sh`
Downloads all Chondrichthyes annotation with reference status in `gff` format to `chondrichthyes_annotations` and generates the file `info_chondrichthyes_annotations.tab` containing accession and species name.
8. `download_teleostei_annotations.sh`
Used to download annotated teleostei genomes with reference status in `gff` format to `teleostei_annoations_ncbi_02/` and to generate the metadata file `info_teleostei_annotations_ncbi_02.tab`.
9. `download_teleostei_assemblies_ncbi_01.sh`
Used to download teleostei assemblies with reference status to `teleostei_assemblies_ncbi_01/` and to extract metadata about each assembly (accession,level, contig-n50, scaffold-n50, organism name, submission date) to `info_teleostei_assemblies_ncbi_01.tab`.
10. `download_teleostei_assemblies_ncbi_02.sh`
Used as `download_teleostei_assemblies_ncbi_01.sh` but for assemblies released after 12/31/2022 to `teleostei_assemblies_ncbi_02/` and metadata file `info_teleostei_assemblies_ncbi_02.tab`.
11. `download_teleostei_assemblies_ncbi_03.sh`
Used as `download_teleostei_assemblies_ncbi_01.sh` but for a set of specified assemblies to `teleostei_assemblies_ncbi_03/`.
12. `download_teleostei_protein_ncbi.sh`
Used to download teleostei protein sequences from reference assemblies to `teleostei_prot_seq_ncbi/` and to extract metadata for each assembly (accession, level, contig-n50, scaffold-n50, organism name, submission date, status, annotation status, type) to `info_teleostei_prot_seq_ncbi.tab`.
13. `info_assemblies.R`
Used to associate assemblies with taxonomic information using assembly metadata files by mapping species names to the taxonomy (`SI_Table_Teleostei_taxonomy.tab`, data file 10). Created `SI_Table_assemblies.tab` (data file 1) containing only the most recent assembly version for each species.

Domain structure analyses

1. `extract_features.sh`
Used to find gff files and run `extract_genes_exons_from_gff.pl` on each of them. Output one file, `<acc>_features.tsv` for each `gff` file, where `<acc>` indicates the accession of the assembly.

   The resulting files were used by `R1/protein_structure.R` and `R3.2/protein_structure.R` and a number of other scripts (see below) that were used to combine domain and intron coordinates in proteins.
2. `count_classes.sh`
Called `count_classes.pl` on all feature files created by `extracted_features.sh`, in order to identify the types of classes commonly used in different gff files.

   Output redirected to `class_counts.tsv`.
3. `count_classes.pl`
Counts the number of entries of each type of class in a feature file. Prints the filename, the class and the number of instances of each class found in the file. Used in order to explore the variation in `gff` formats.
4. `extract_genes_exons_from_gff.pl`
Converts a `gff` file to a tab delimited format with the following columns:
   1. class: The class of the feature (eg., region, gene, mRNA, CDS)
   2. id
   3. parent: the id of the parent; if no parent NA
   4. name
   5. product
   6. description
   7. note

8. chr
9. strand
10. frame
11. start
12. end
13. source

Many of these fields are taken from the description field of the gff and are present only for some features. If not present `NA` is output.

5. `identify_prdm9_genes.sh`
   Called `identify_gene_candidates.pl` on all feature files output by `extract_features.sh`.

   Output a single file: `prdm9_candidates.tsv` by redirection of `STDOUT`.

6. `identify_gene_candidates.pl`
   Searches the `gene` and `mRNA` features in feature files produced by `extract_genes_exons_from_gff.pl` for matches to a specified regular expression. Outputs tab separated values to `STDOUT`:

   1. The name of the file parsed
   2. The class of feature (eg. mRNA, gene)
   3. The parent id (if mRNA, this will be the gene id)
   4. The feature id
   5. The types of annotation containing a match (comma separated)
   6. The matching texts comma separated.

7. `non_teleosts/extract_features.sh`
   Wrapper script used to run `extract_genes_exons_from_gff.pl` on gff files in `../annotated_genomes_non_teleosts/` in order to extract gene, transcript, exon and coding sequence coordinates from a selected set of non-teleost species.

8. `non_teleosts/extract_genes_exons_from_gff.pl`
   This is a copy of `extract_genes_exons_from_gff.pl` used by `non_teleosts/extract_features.sh`. It was used to create feature files from gff files from a set of non-teleost species.

9. `non_teleosts/identify_prdm9_genes.sh`
   Wrapper script used to run `identify_gene_candidates.pl` on feature files produced by `non_teleosts/extract_features.sh`.

10. `list_ps_scan_files.sh`
    Wrapper of a `find` command used to list the output of interproscan output files. Was used to create the `ps_scan_files.txt` used by several scripts to find both protein sequence and domain scan files.

11. `R1/tel_interpro_scans.sh`
    Ran `interproscan.sh` (the interproscan shell wrapper) on teleost candidate PRDM9 amino acid sequences extracted from assemblies and translated in `teleost_prdm9_pep.faa` by `R1/protein_structure.R`. Output files in `.tsv` and `.gff3` for each of the databases used (Pfam SUPERFAMILY Gene3D ProSiteProfiles PRINTS Coils).

12. `R1/nt_interpro_scans.sh`
    As `R1/tel_interpro_scans.sh`, but on sequences from non-teleost vertebrates.

13. `prosite_hmmer_scans_2/parse_domain_scans.sh`
    Called `prosite_hmmer_scans_2/parse_domain_scans.pl` to parse both the output of `pfscanV3` and `hmmsearch` run on teleost proteomes. Output several files, one of which contains both domain matches, protein lengths and domain structures for all protein sequences scanned. Output produced was used as input for `R3.2/protein_structure.R`.

    1. `teleost_domain_introns.tsv`
       Positions of domains and introns in protein sequences.
    2. `teleost_cds_gene.tsv`
       Mapping between protein and gene identifiers.
    3. `teleost_cds_mRNA.tsv`
       Mapping between protein and mRNA identifiers.
    4. `teleost_features_sel.tsv`
       CDS features from selected genes and transcripts.

14. `run_hmmer_aKRAB-A.sh`
    Scan of teleost protein sequences for the aKRAB domain.

15. `run_hmmer_ePRSET_2.sh`
    Scan of teleost proteins sequences for the ePR-SET domain.

16. `run_prosite_PRDM9.sh`
    Ran `pfscanvV3` on teleost protein sequences.

17. `prosite_hmmer_scans_2/parse_domain_scans.pl`
    Parses `pfscanV3` and `hmmsearch` output to a common tabular format and integrates the matches with the positions of introns within protein sequences.
18. `prosite_hmmer_scans_2/count_overlap.pl`
    Script used to determine the reasons for the difference in the number of assemblies for which we had protein sequences and `gff` annotation. These differences were due to the lack of synchronisation in the download of data.
19. `HMM_PRSET_seq_2/extract_hmm_set.pl`
    Extracted sequences matching the ePR-SET domain from proteomes obtained from NCBI to `hmm_set_domains.fa`. These sequences were used by `R3.2/protein_structure.R` for the multiple sequence alignment leading to data shown in Figure 2.
20. `R1/protein_structure.R`
    Analyses of teleost protein sequences annotated as PRDM9 or related to PRDM9. These were used to define the ePR-SET HMM and for supplementary figures 2-4.
21. `R1/functions.R`
    Functions for `R1/protein_structure.R`.
22. `R3.2/protein_structure.R`
    Analyses of protein sequences with matches to several PrositeProfiles and hidden Markov models. These analyses led to figures 2 and 3 and supplementary figures 5-13.
23. `R3.2/functions.R`
    Functions for `R3.2/protein_structure.R`.
24. `hmmer_profiles/build_hmm_PRSET_teleostei.sh`
    Script used to build a hmm profile of an extended ePR-SET domain from a multiple sequence alignment of teleost PRDM9 SET domain with flanking sequences.

Intron sizes

1. `Teleostei_IntronSizes_GenBank.sh`
   Used to extract intron lengths from unique transcripts in GenBank (GCA) gff files. Output results in bed format containing chromosome/scaffold, start and end position of intron, gene name, strand and length of intron.
2. `Teleostei_IntronSizes_RefSeq.sh`
   Used to extract intron lengths from unique transcripts in RefSeq (GCF) gff files. Output results in bed format containing chromosome/scaffold, start and end position of intron, gene name, strand and length of intron.
3. `vertebrate_intron_distributions.R`

4. `intron_size_ncbi.R`
   Used to Generate a summary table of intron sizes including taxonomic information. Used annotation information file `info_teleostei_annotations_ncbi_02.tab` to map species names to their taxonomy (`SI_Table_Teleostei_taxonomy.tab`). Output `SI_Table_IntronSizes.tab`.
5. `intron_size_braker.R`
   Generated a summary table of intron sizes including taxonomic information for assemblies annotated here. Used assembly information files to map species names to their taxonomy (`SI_Table_Teleostei_taxonomy.tab`). Output `SI_Table_IntronSizes_braker.tab` containing intron size information for all species annotated in this study.
6. `Teleostei_IntronSizes_braker.sh`
   Used to extract intron lengths from unique transcripts of the annotation files generated in this study. Output results in a bed format containing chromosome/scaffold, start and end position of intron, gene name, strand and length of intron.
7. `label_by_accession.sh`
   Labels bed files containing intron sizes by the folder name (=accession) they are contained in.
8. `intron_distributions_manual_classification/manual_annotation.R`
   Read intron sizes from `SI_Table_IntronSizes.tab` and determined intron size distributions from all species. The order of the distributions were randomised, plotted and classified interactively by LMJ in the absence of any taxonomic information. The classification was then used to label points in Fig. S19.

aKRAB in genomic sequences

1. `extract_orfs.pl`
   Extracts all open reading frames in six frames from nucleic acid sequences. Takes a sequence file as input and outputs to STDOUT.
2. `hmmer_aKRAB.sh`
   Used to scan genomic sequences for the aKRAB HMM by translating the six frames using `extract_orfs.pl` and then piping the output to `hmmserach`. Output to hmmer standard output format, tabular output and stockholm alignment.
3. `parse_hmmer.R`
   Used to combine `hmmsearch` output with taxonomic information (species, order, clade, family). Output SI_Table_aKRAB_hmmer.tab.
4. `download_sra.sh`
   Downloaded raw sequencing runs of accessions listed in `tblastn_accessions.tab` with prefetch. Prefetched runs in compressed SRA format were converted to fasta and fastq format with fasterq-dump (–fasta) and fastq-dump, respectively.
5. `something to run bwa`
   Script or details of the run.
6. `cov_expected.sh`
   Calculates average coverage of sorted bam files with samtools depth and awk. Prints 'Average =' to coverage.txt.
7. `make_blastdb.sh`
   Used to build a blast database using fasta files as input obtained using `download_sra.sh`..
8. `run_tblastn.sh`
   Used to align the first 250 amino acids of *G.morhua* PRDM9 orthologue (XP_030231029.1) to sequences obtained using `download_sra.sh` using tblastn. Output results in tabular format for all reads.

Supplementary Figures

List of Figures

S1 Teleost intron size distributions. 16
S2 Numbers of PRDM9 orthologue candidates 17
S3 Domain structure of PRDM9 orthologue candidates 18
S4 Domain structures of PRDM9 orthologue candidates 19
S5 Protein domain combinations 20
S6 SET and aKRAB scores 21
S7 Protein groups defined by two SET domain models 22
S8 Mouse and human ePR-SET scores 23
S9 Avian ePR-SET scores 23
S10 Domain structure of PRDM9 orthologue candidates (2) 24
S11 Numbers of PRDM9 orthologue candidates 25
S12 Domain structures of PRDM9 orthologue classes 26
S13 Domain sequence distance distributions 27
S14 aKRAB single best one domain score. 28
S15 aKRAB losses in Osteoglossiformes. 29
S16 aKRAB loss in Clupeiformes. 30
S17 Coverage plots of tblastn search. 31
S18 aKRAB distribution in Gadiformes. 32
S19 Distinct distributions of teleost intron sizes. 33

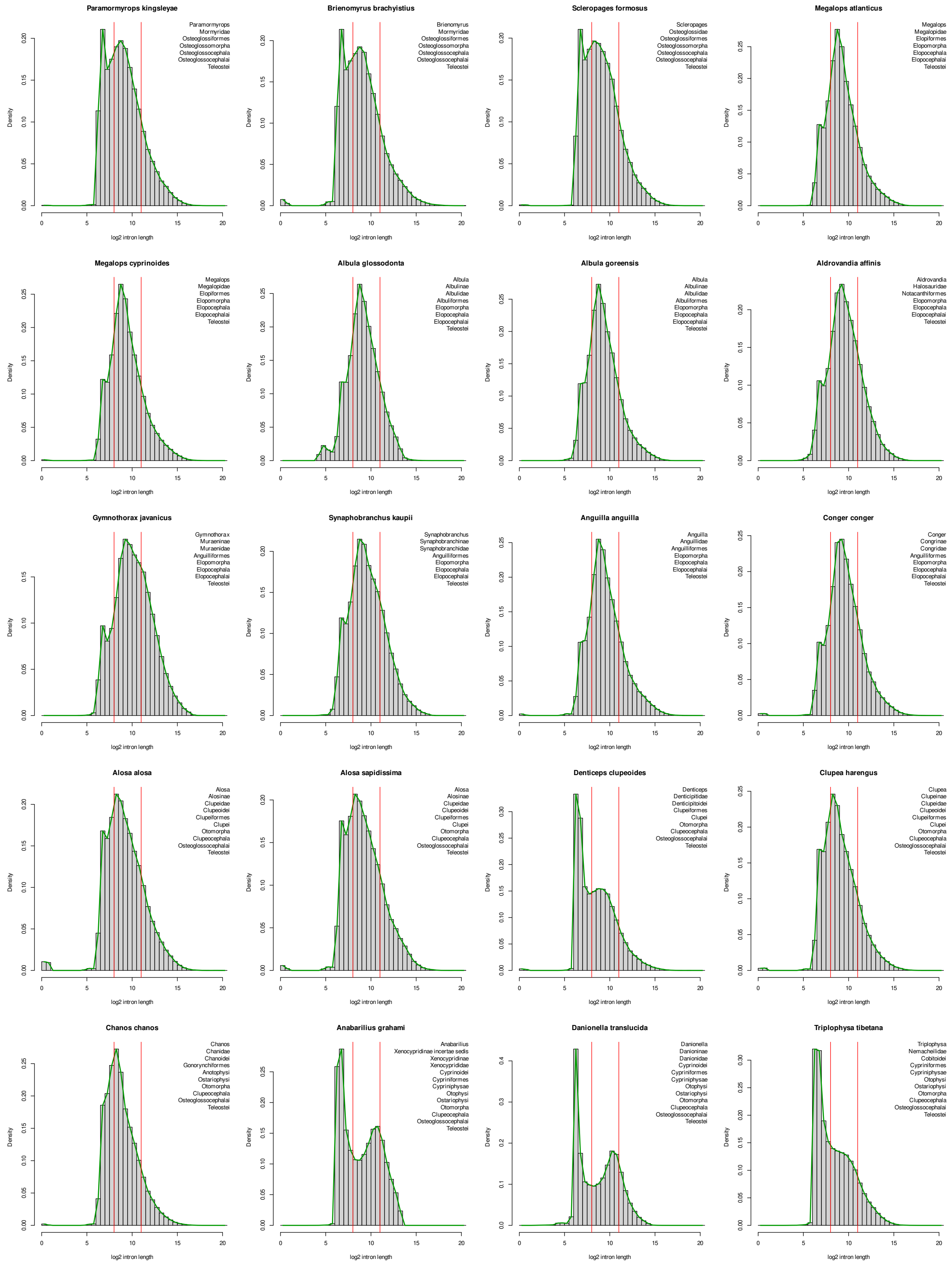

Fig. S1 Teleost intron size distributions.
log2 transformed intron size distributions of teleosts determined from gene annotations available from RefSeq and GenBank submissions. Taxonomic identification is taken from the NCBI taxonomy and shown in the top right corner for each species. The two red vertical lines indicate antimode position and peak of long intron sizes in *Danio rerio*. This shows the first page of the full set of distributions which are given in supplementary file 2.

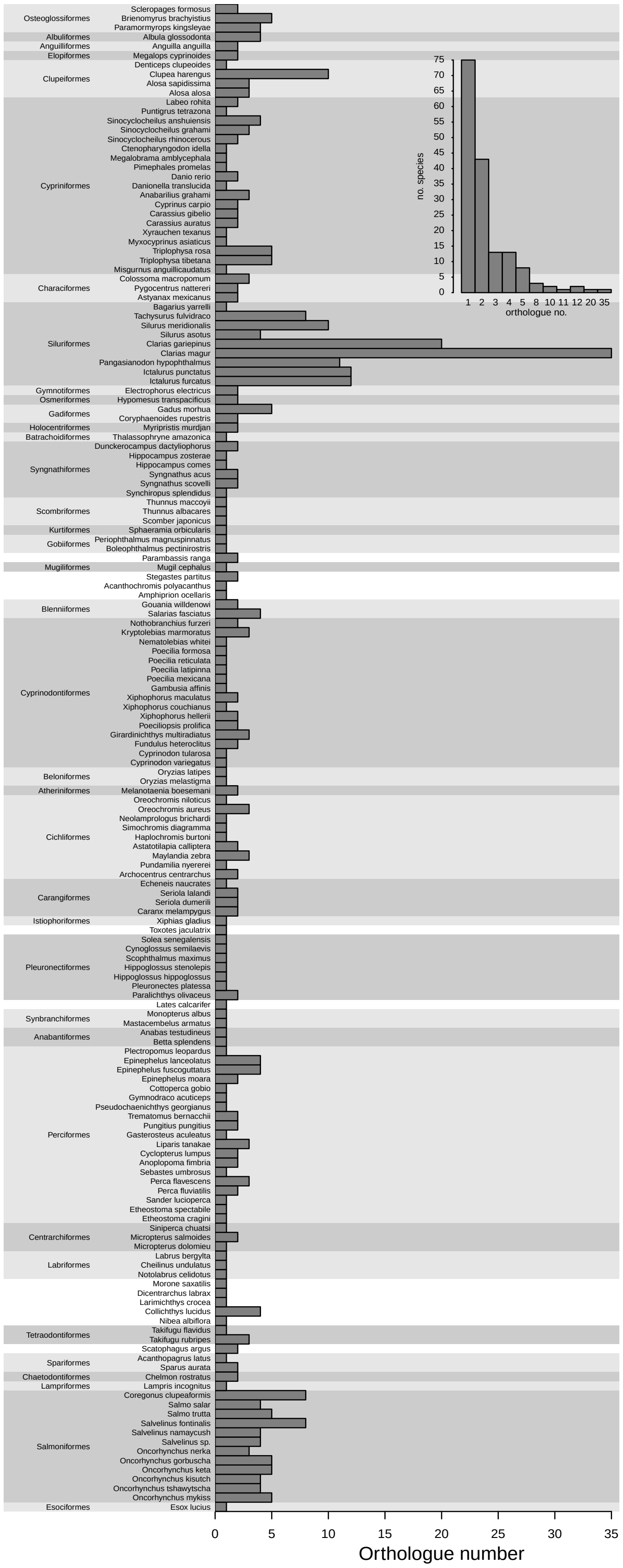

Fig. S2 Numbers of PRDM9 orthologue candidates
Number of genes annotated with the term “PRDM9” (case insensitive) per species. Species are grouped by taxonomic orders shown in the left-most panel. Inset shows the counts of species with different numbers of orthologue counts. 75 species had a single and one species had 35 candidates.

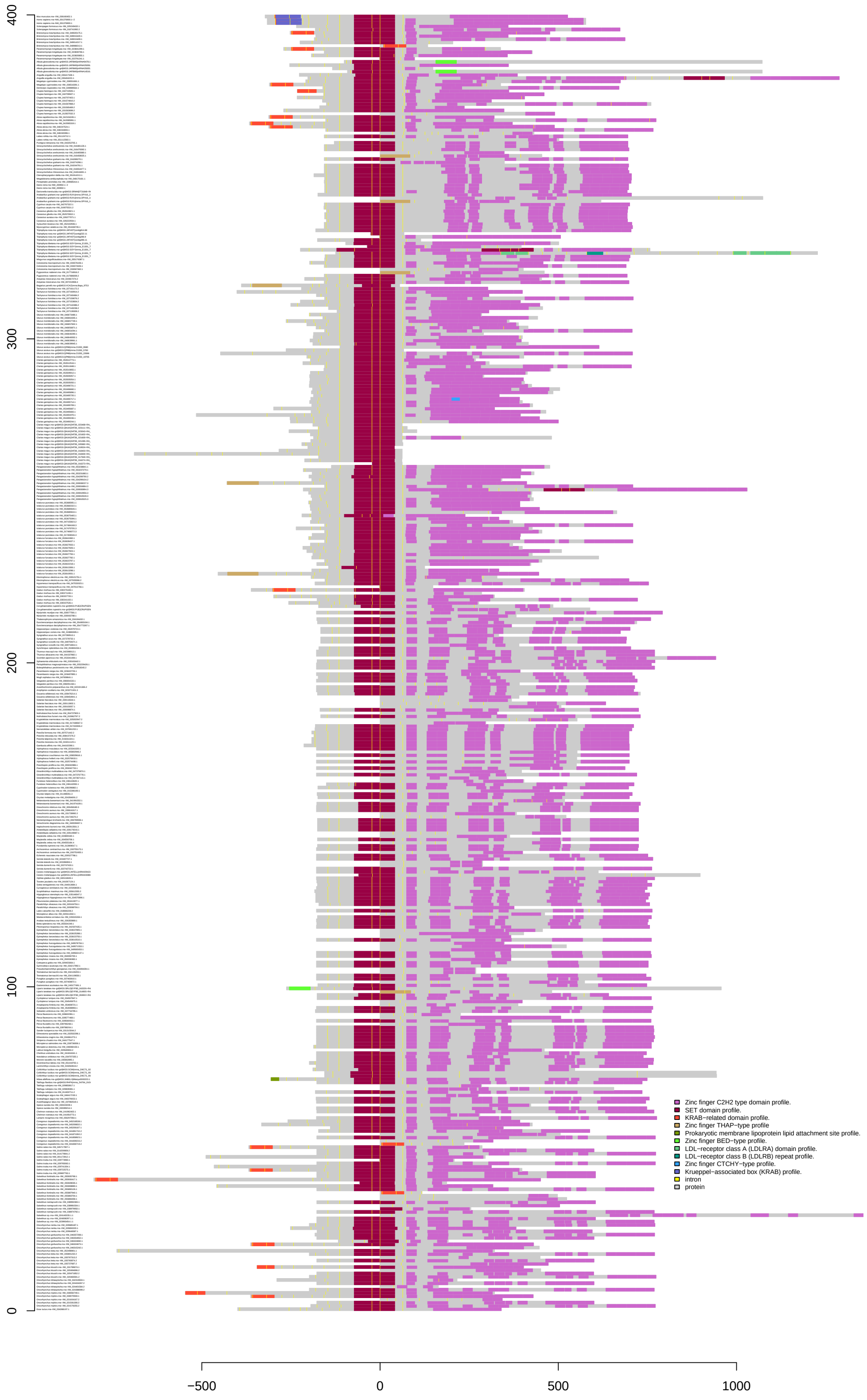

Fig. S3 Domain structure of PRDM9 orthologue candidates
Proteins are shown as grey rectangles overlaid with coloured rectangles representing matches to PrositeProfiles domains. Yellow lines indicate the position of introns. The proteins have been aligned by the position of the last intron in the SET-domain and are ordered by taxonomic order as in Fig. S2. The top three rows show human and mouse protein structures.

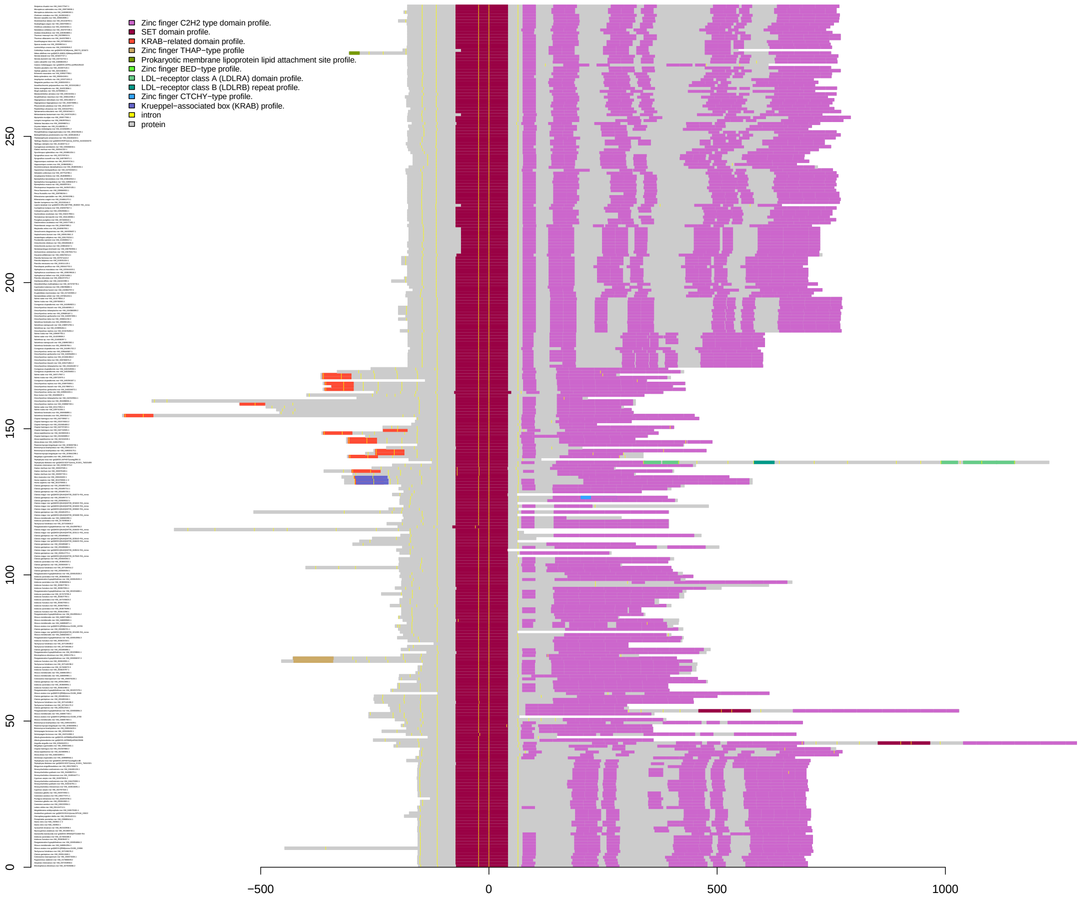

Fig. S4 Domain structures of PRDM9 orthologue candidates
Candidate orthologues with SET domains ordered by a multiple sequence alignment of extended SET domains from the exons containing the SET domains. Mouse and human sequences can be recognised as the only ones with matches to the Krueppel-associated box profile (dark blue). Proteins are shown as grey rectangles overlaid with coloured rectangles representing matches to PrositeProfiles domains. Yellow lines indicate the position of introns.

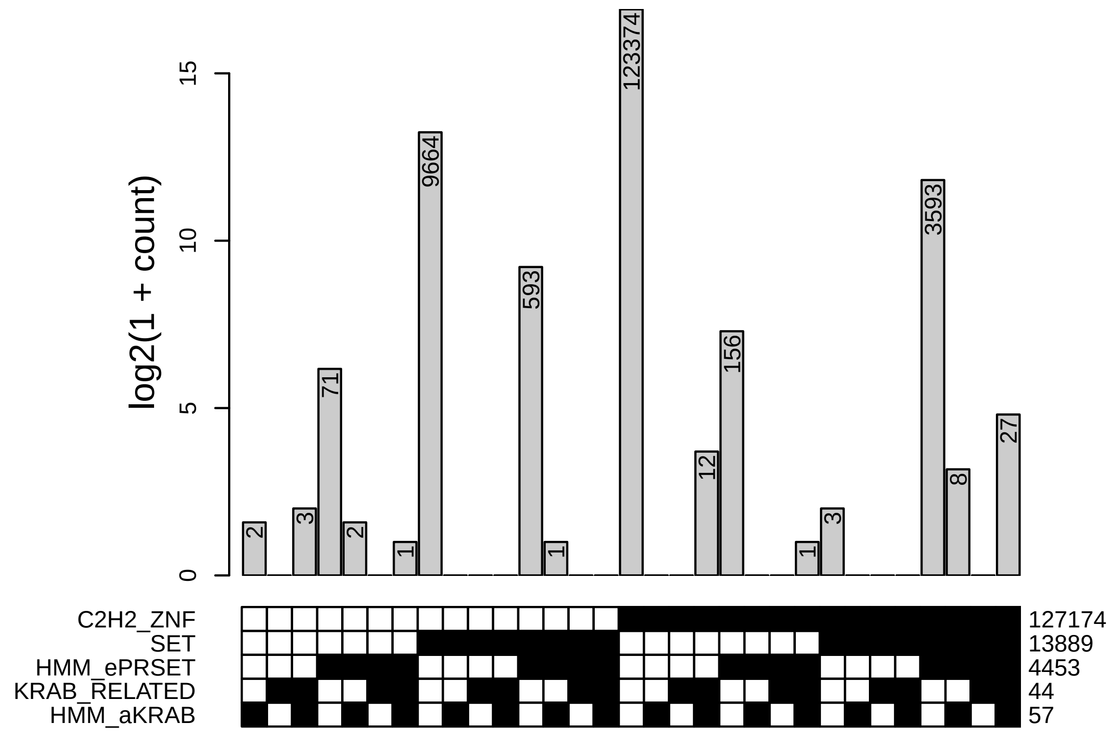

Fig. S5 Protein domain combinations
The number of genes encoding proteins with the indicated combination of domains shown to the left of the lower panel.
Upper panel: number of proteins with the domain content specified in the lower panel. Note that the counts are $\log_2$ transformed and that the actual number of genes is shown at the top of each bar.
Lower panel: Black squares indicate domain combinations; eg. the first three columns indicate proteins with matches to *only* 1) HMM_aKRAB, 2) KRAB_RELATED and 3) both HMM_aKRAB and KRAB_RELATED domains respectively. That is, two genes encoded proteins with matches to *only* the HMM_aKRAB domain and three genes encoded matches to *only* HMM_aKRAB *and* KRAB_RELATED profiles. Numbers to the right show the total number of genes with matches to the domains indicated.

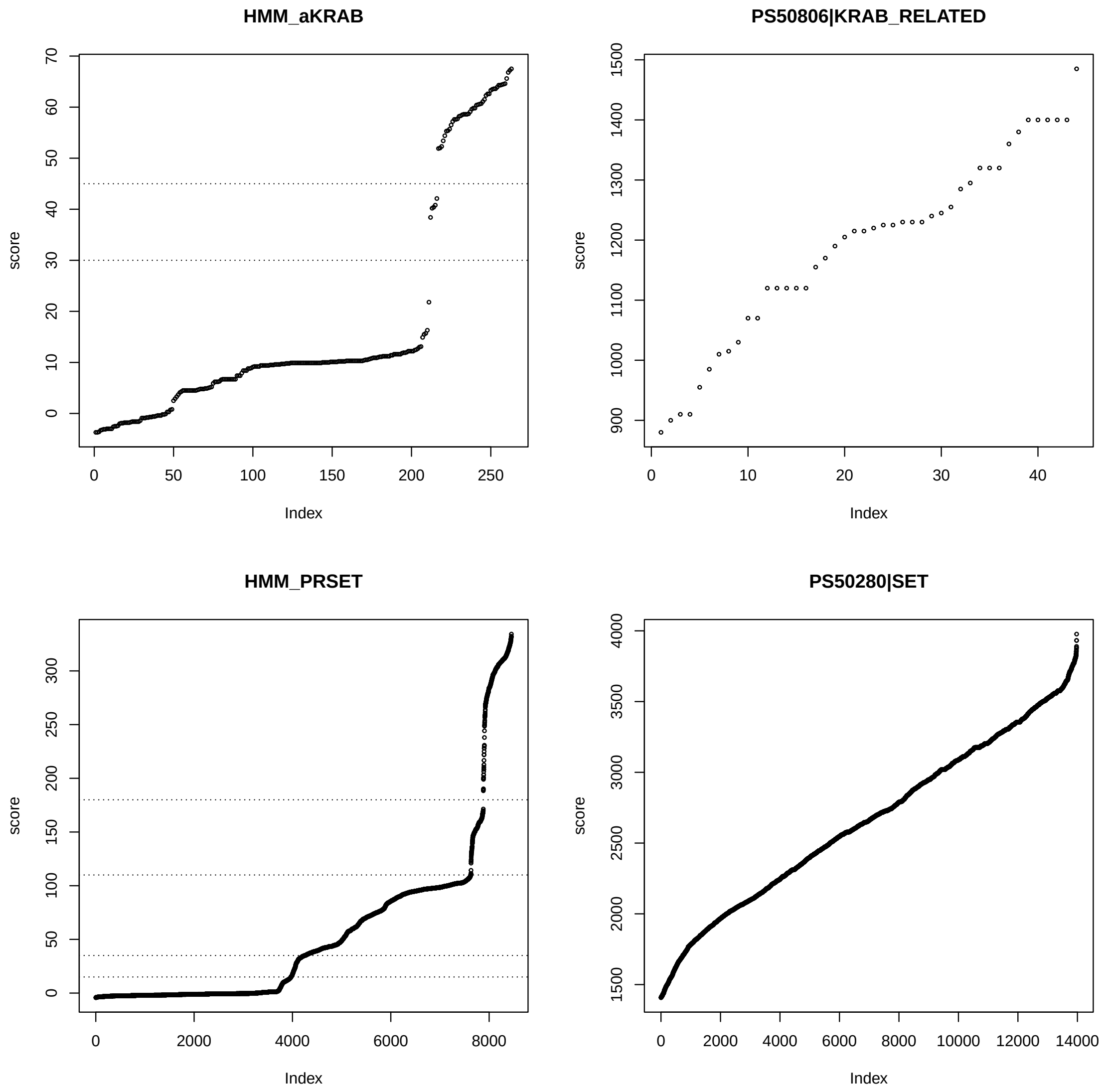

Fig. S6 SET and aKRAB scores
Quantile plots of scores for different models for the aKRAB and SET domains obtained by scanning proteins from 185 different teleost species. Scores have been sorted from low to high. Left hand plots show scors obtained by running hmmer with the indicated HMMs. Note that HMM_PRSET was generated by us (ePR-SET). Right hand plots show scores obtained for PrositeProfiles models.

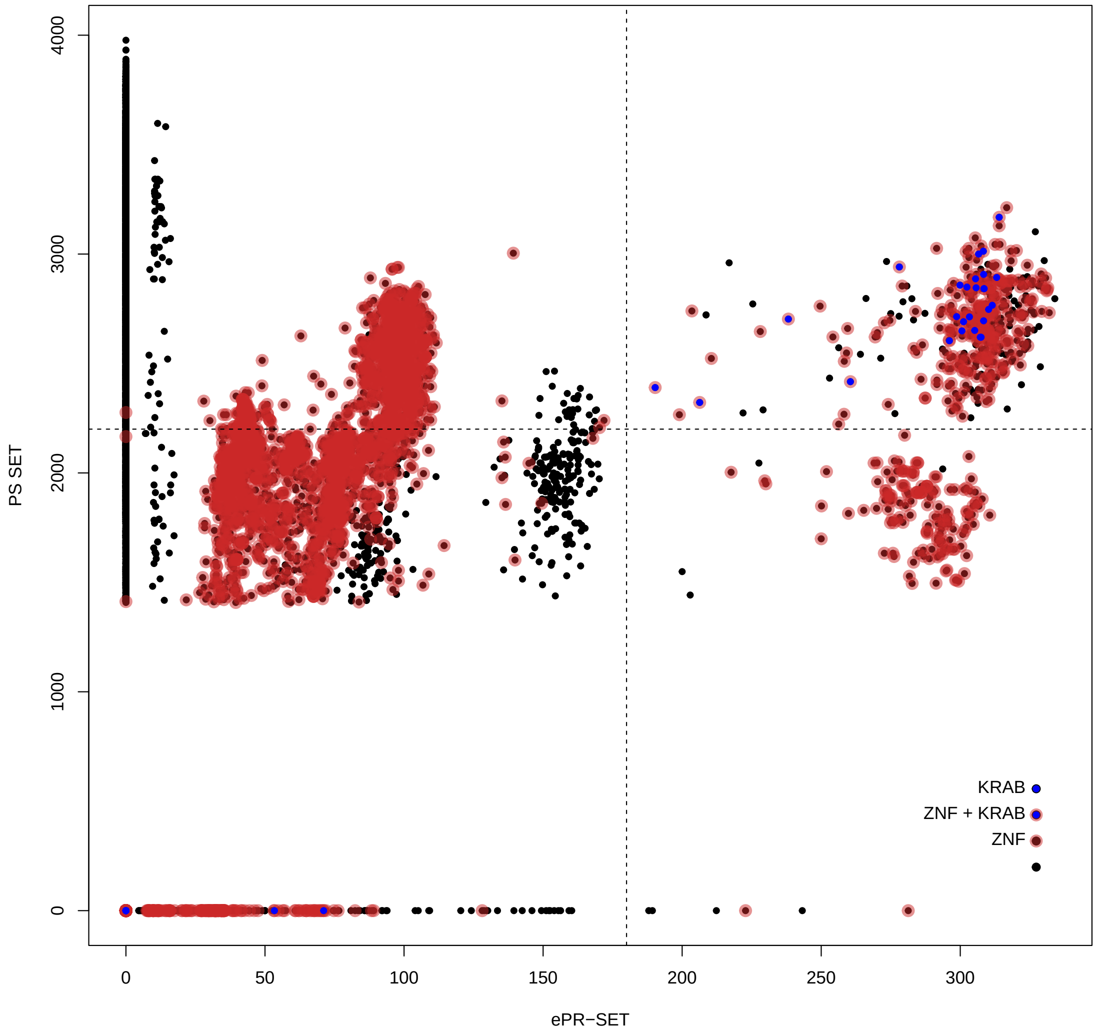

Fig. S7 Protein groups defined by two SET domain models
Domain alignment scores obtained by an HMM model against an extended PR-SET domain (x-axis) and the PrositeProfiles SET domain (y-axis). Each point represents one protein; the presence of matches to the aKRAB and C2H2 zinc fingers (ZNF) in each protein is indicated by colour. The combination of scores defines a number of clearly defined clusters that correlate with the presence of other domains.

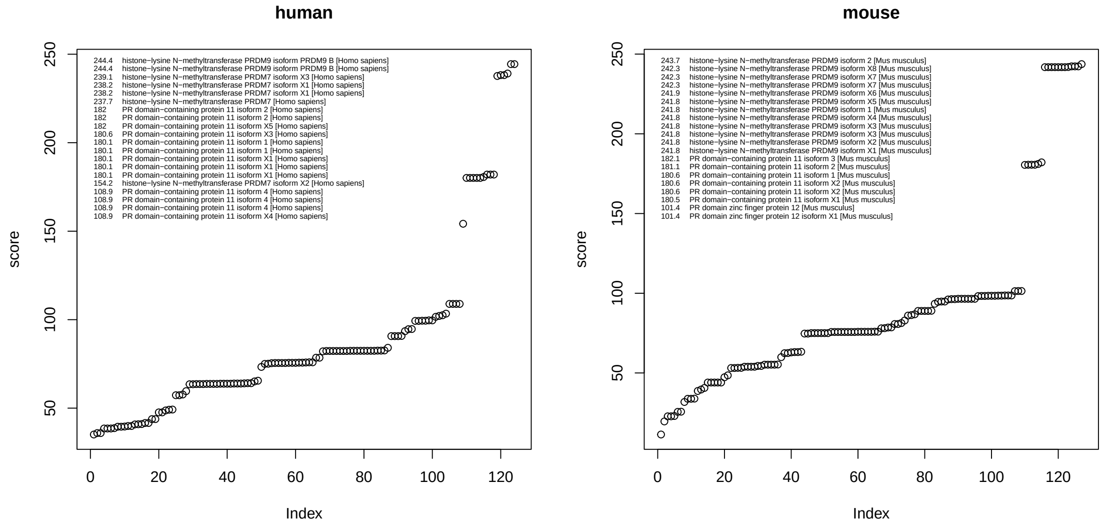

Fig. S8 Mouse and human ePR-SET scores
Quantile plots of scores for matches to the ePR-SET HMM in human and mouse sequences. The description of proteins with the top scores are shown with the actual score on the left.

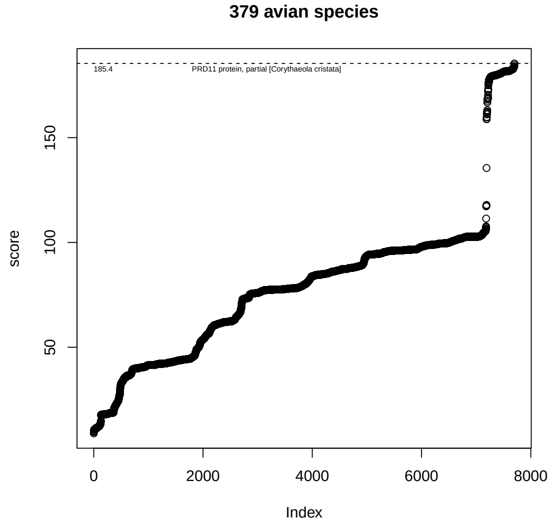

Fig. S9 Avian ePR-SET scores
Quantile plots of scores for matches to the ePR-SET HMM in protein sequences obtained from 279 avian species. The annotation for the top scoring protein is shown.

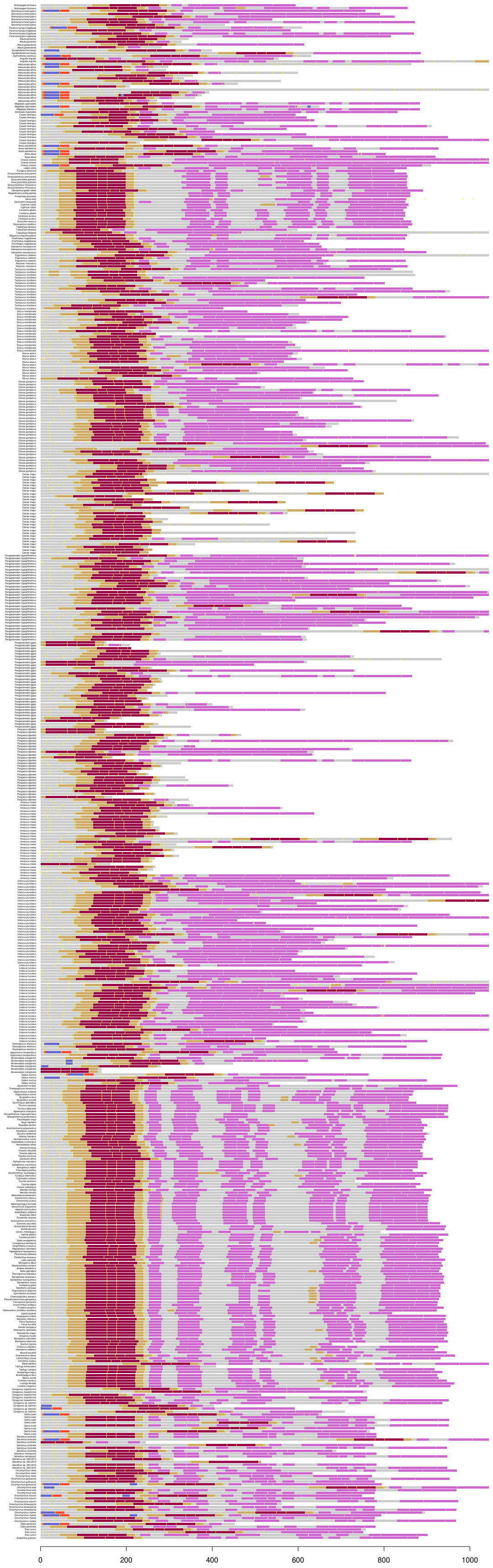

Fig. S10 Domain structure of PRDM9 orthologue candidates (2)
Domain structures of teleost proteins with matches to the ePR-SET HMM with scores higher than 180. Proteins are shown as grey rectangles overlaid with coloured rectangles representing matches to PrositeProfiles domains. Yellow lines indicate the position of introns. Proteins are arranged by species taxonomic order. Domains are indicated by colours (see legend in Fig. S12).

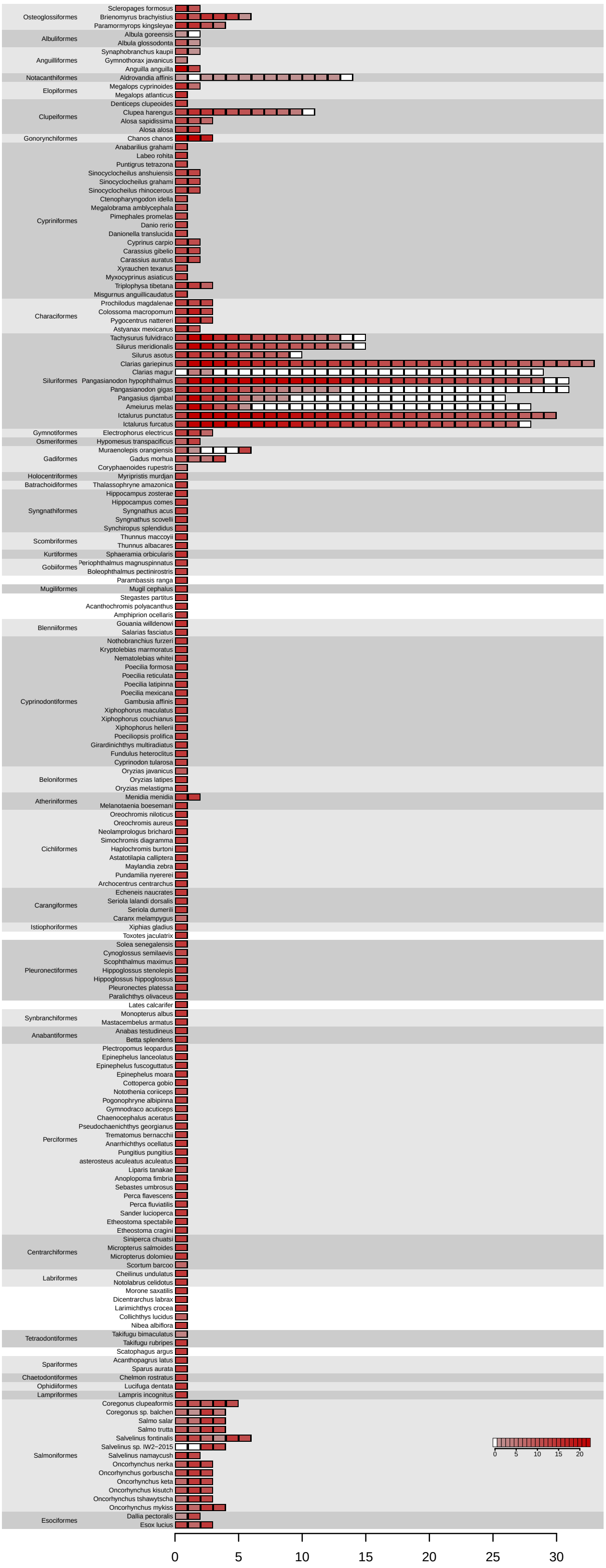

Fig. S11 Numbers of PRDM9 orthologue candidates

Numbers of proteins per species with matches scoring more than 180 for the ePR-SET HMM. The numbers of proteins are indicated as stacked rectangles, where each rectangle represents a single protein. Red shading indicates the number of Zn-finger domains in each protein as indicated by the scale. Note that the scale goes to 22, but a number of proteins contained far more Zn-finger domains.

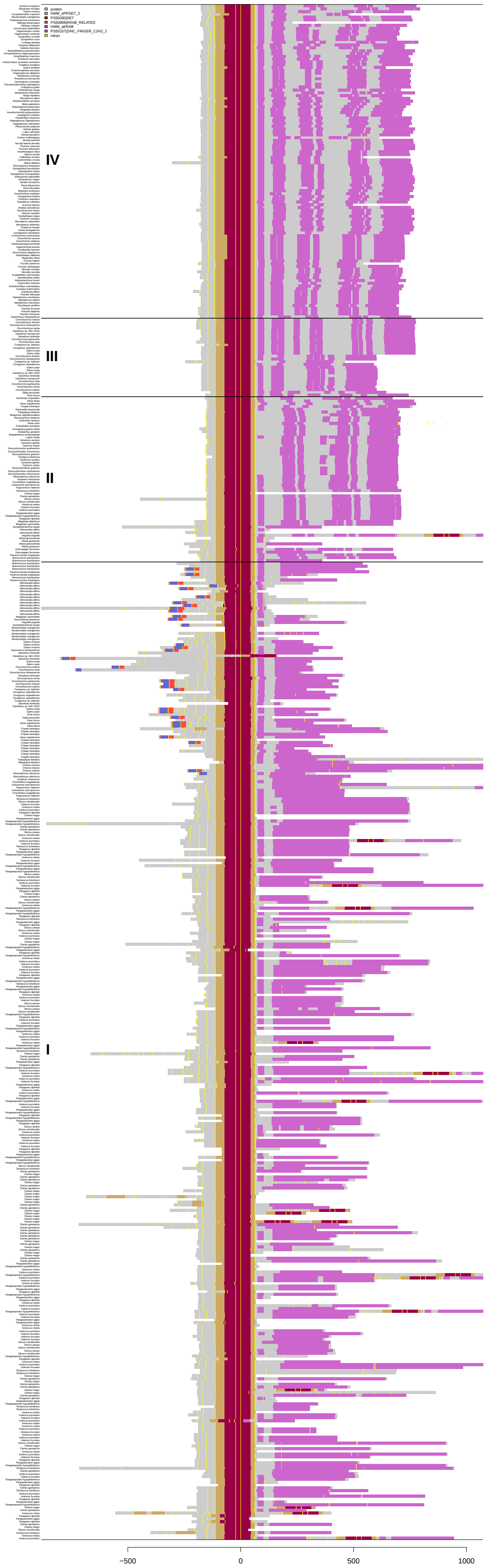

Fig. S12 Domain structures of PRDM9 orthologue classes
The sequences matching the ePR-SET model with scores above 180 were aligned and used to define distinct classes of ePR-SET sequences and an ordering based on neighbour-joining tree based on pairwise distances. The proteins, domain structures and introns are represented by rectangles as in figures S10, S4 and S3. The domain positions were aligned by the second intron of the SET domain. Domain models used are shown in the legend. Note that HMM_ePRSET_2 refers to the ePR-SET model created in this study.

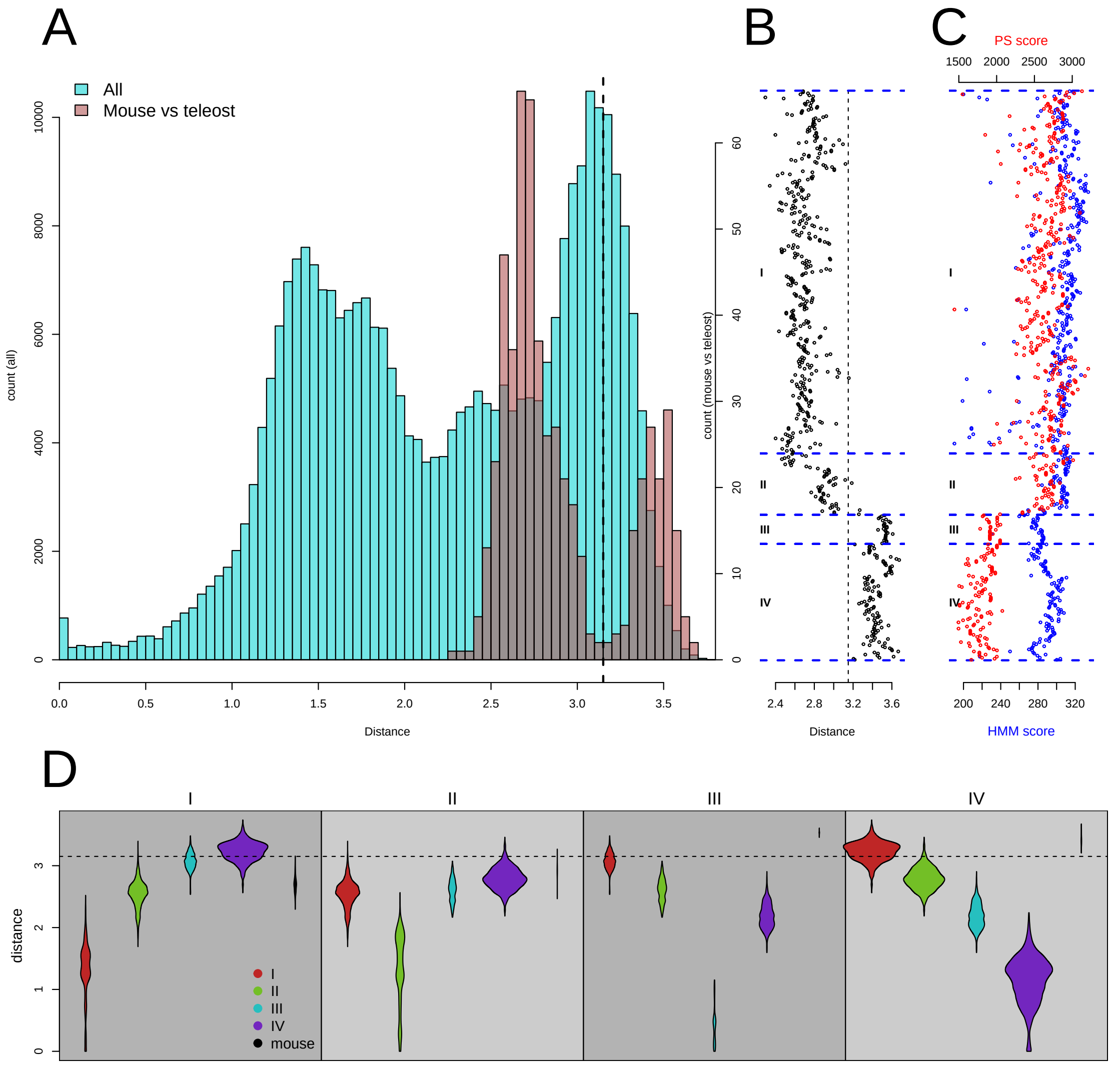

Fig. S13 Domain sequence distance distributions
A. The distribution of distances between sequences matching to the ePR-SET HMM. Distributions are shown separately for all pairwise (All) and distances between the matching mouse sequence and all teleost sequences. B. Distances between mouse and teleost sequences ordered by a neighbour joining tree as in Fig. S12. C, Scores for matches to the PrositeProfiles SET domain model and the ePR-SET HMM ordered as in B. D. Distributions of distances between proteins of different classes; left-most panel shows distances between class I and itself (red) and the other classes (green, cyan, purple) as well as the mouse sequences. Note that the distances between class I and class IV are larger than those between class I and mouse sequences.

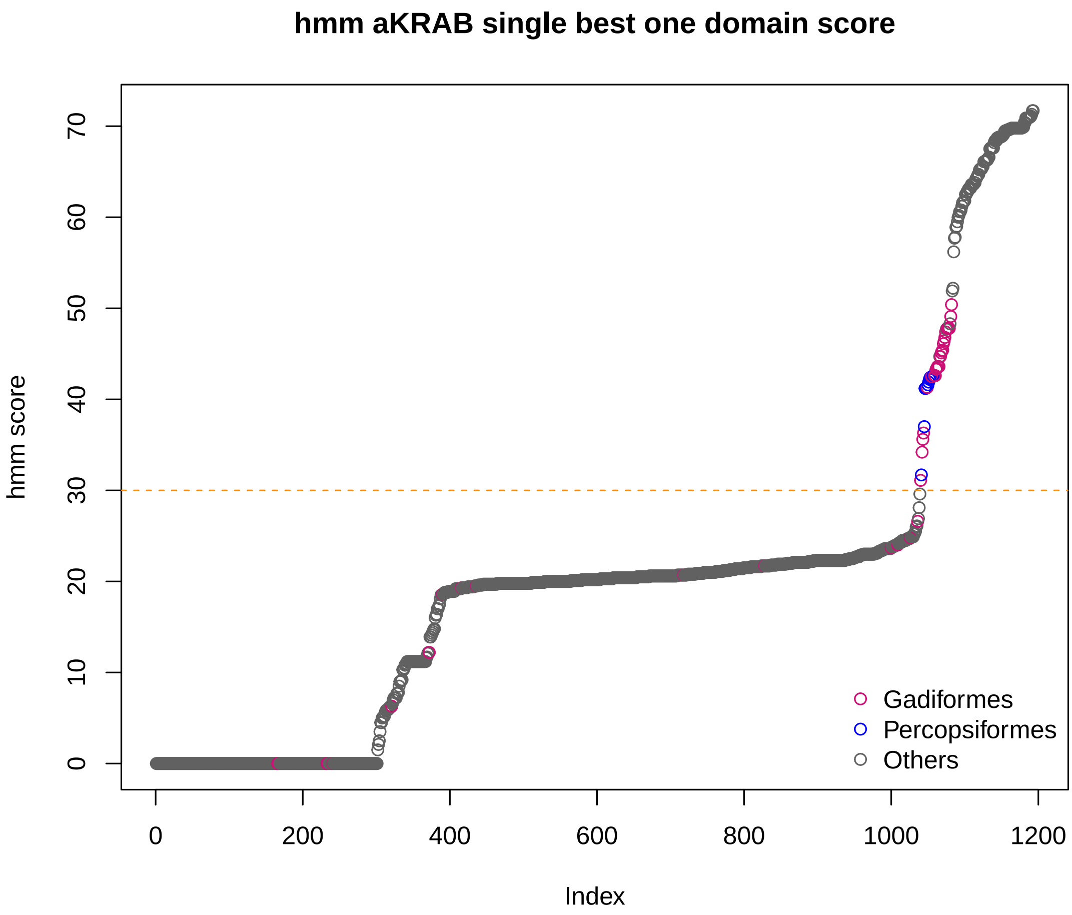

Fig. S14 aKRAB single best one domain score.
Highest hmmer one domain score of teleost species resulting from the hmmer search with an aKRAB-A specific HMM against the genome assemblies. Scores of gadiform and percopsiform species are highlighted in pink and blue, respectively. The orange vertical line at a score of 30 represents the cutoff between presence and absence of aKRAB.

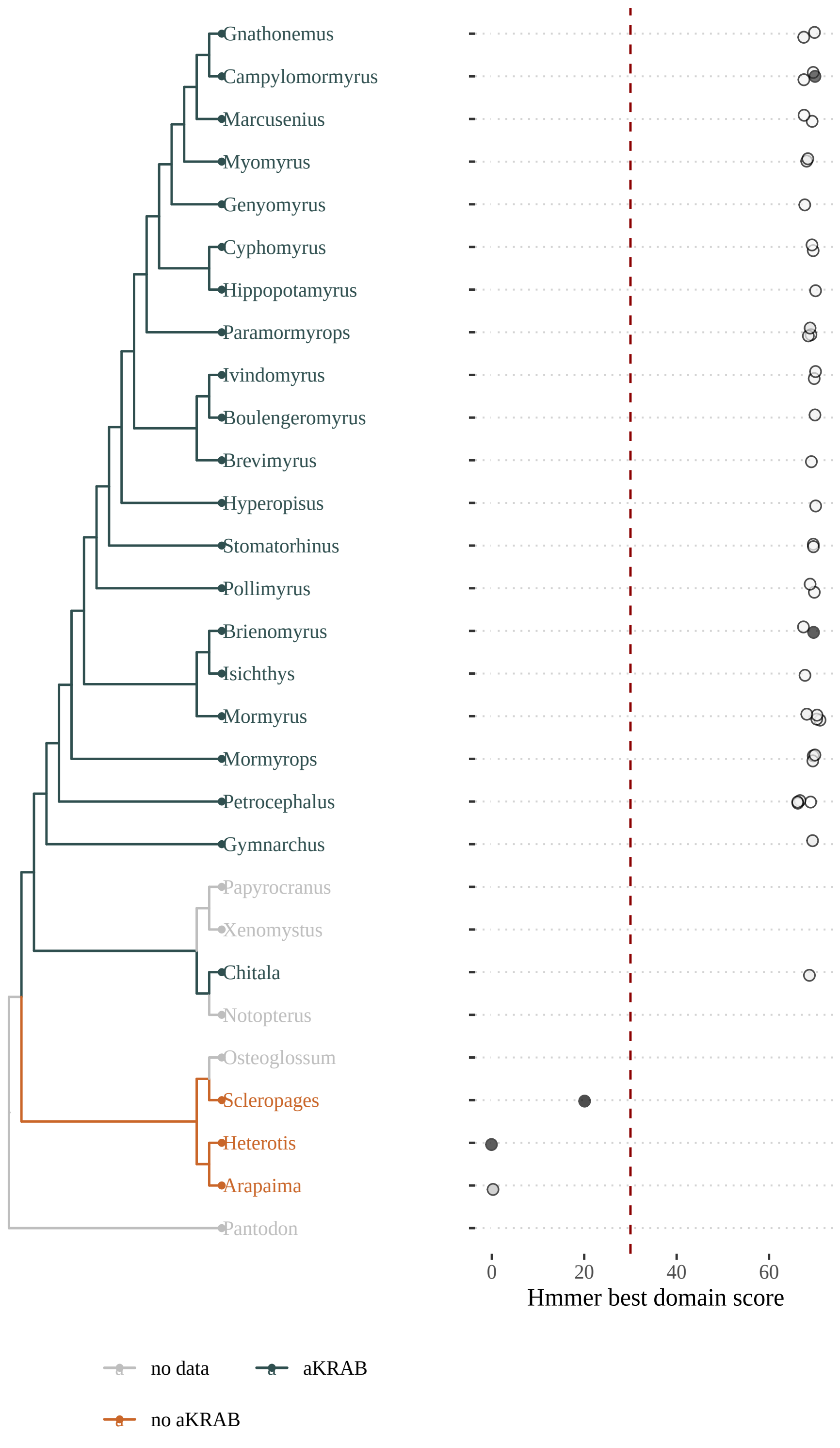

Fig. S15 aKRAB losses in Osteoglossiformes.
Phylogenetic tree of genera within the order Osteoglossiformes and associated hmmer best one domain score. Points are colored by $\log_{10}$ contig N50 of respective genome assembly. Red vertical line at a score of 30 denotes threshold between presence and absence of aKRAB domain.

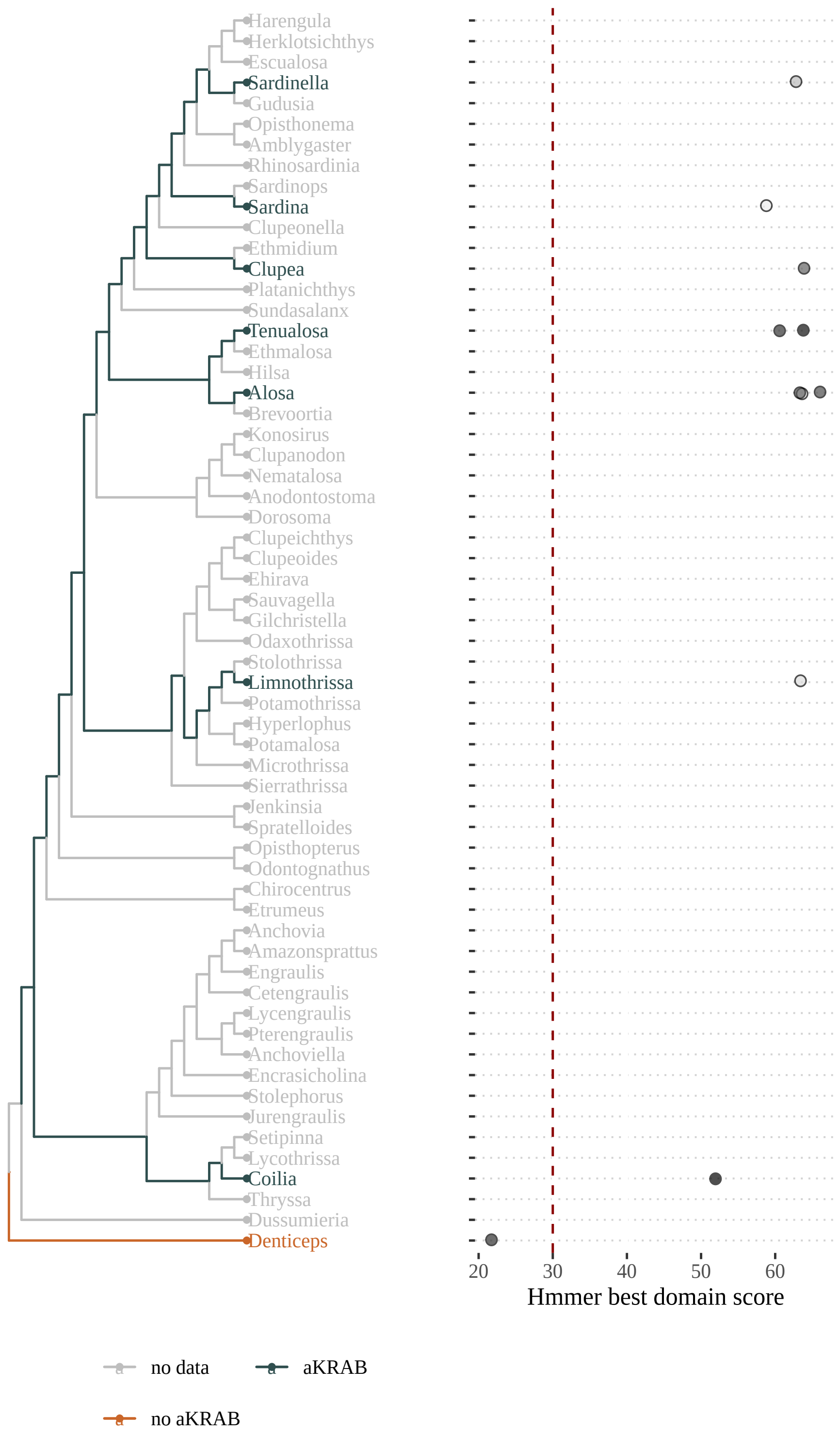

Fig. S16 aKRAB loss in Clupeiformes.
Phylogenetic tree of genera within the order Clupeiformes and associated hmmer best one domain score. Points are colored by $\log_{10}$ contig N50 of respective genome assembly. Red vertical line at a score of 30 denotes threshold between presence and absence of aKRAB domain.

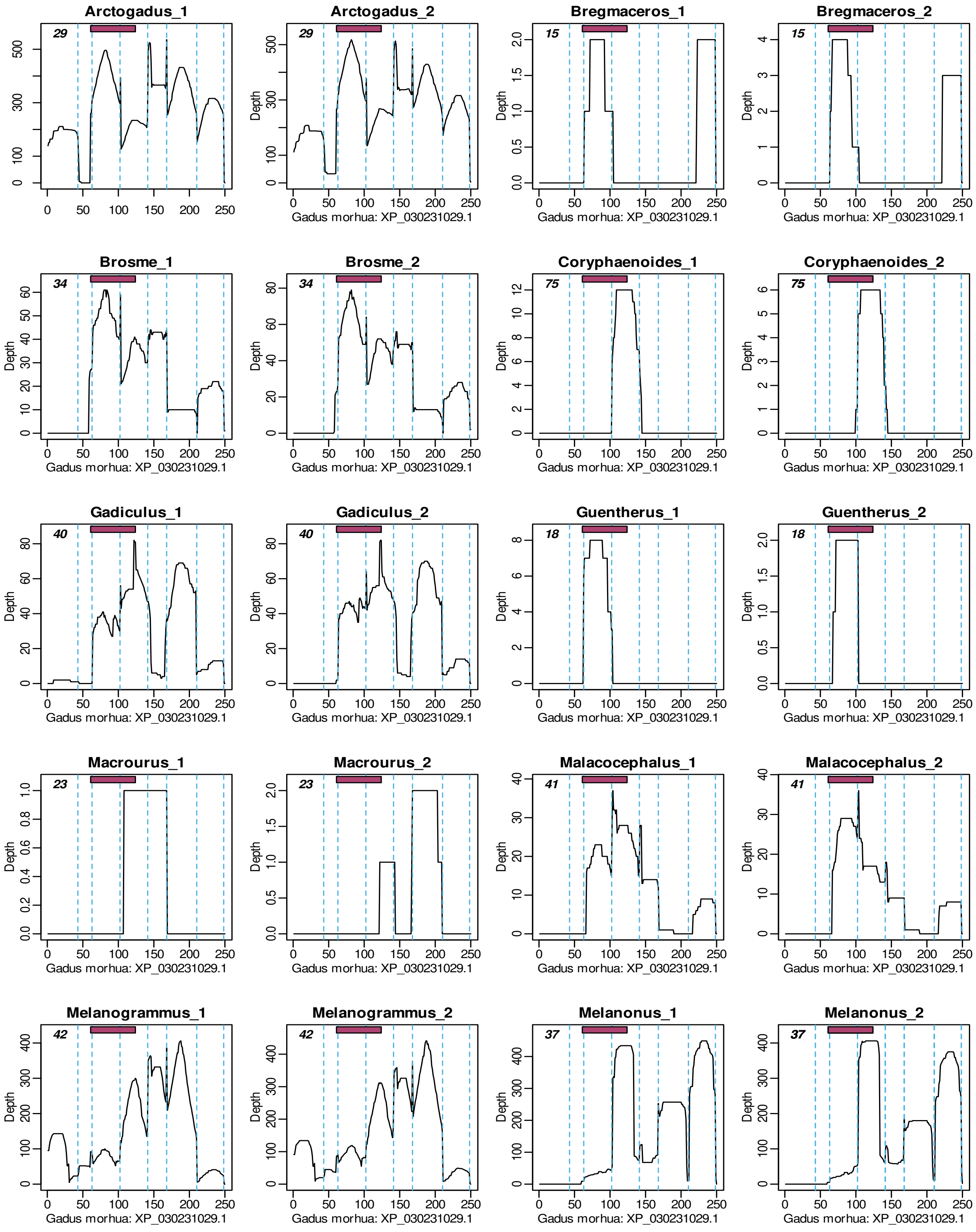

Fig. S17 Coverage plots of tblastn search.
Number of reads overlapping each position of *Gadus morhua* PRDM9 (XP_030231029.1). Red rectangle shows aKRAB domain in XP_030231029.1. Suffixes denote forward and reverse reads, respectively. Blue lines indicate position of introns in *G.morhua*. Number in upper left corner shows average depth. *Arctogadus glacialis* (ERR1473882), *Bregmaceros cantori* (ERR1473845), *Brosme brosme* (ERR1473875), *Coryphaenoides rupestris* (SRR6354872), *Gadiculus argenteus* (ERR1473877), *Macrourus berglax* (ERR1473864), *Malacocephalus occidentalis* (ERR1473866), *Melanogrammus aeglefinus* (ERR2028455), *Melanonus zugmayeri* (ERR1473851), *Cyttopsis rosea* (ERR1473843), *Zeus faber* (ERR11641144), *Guentherus altivela* (ERR1473837). Note that *Z.faber* and *C.rosea* are not displayed as they did not yield any hits.

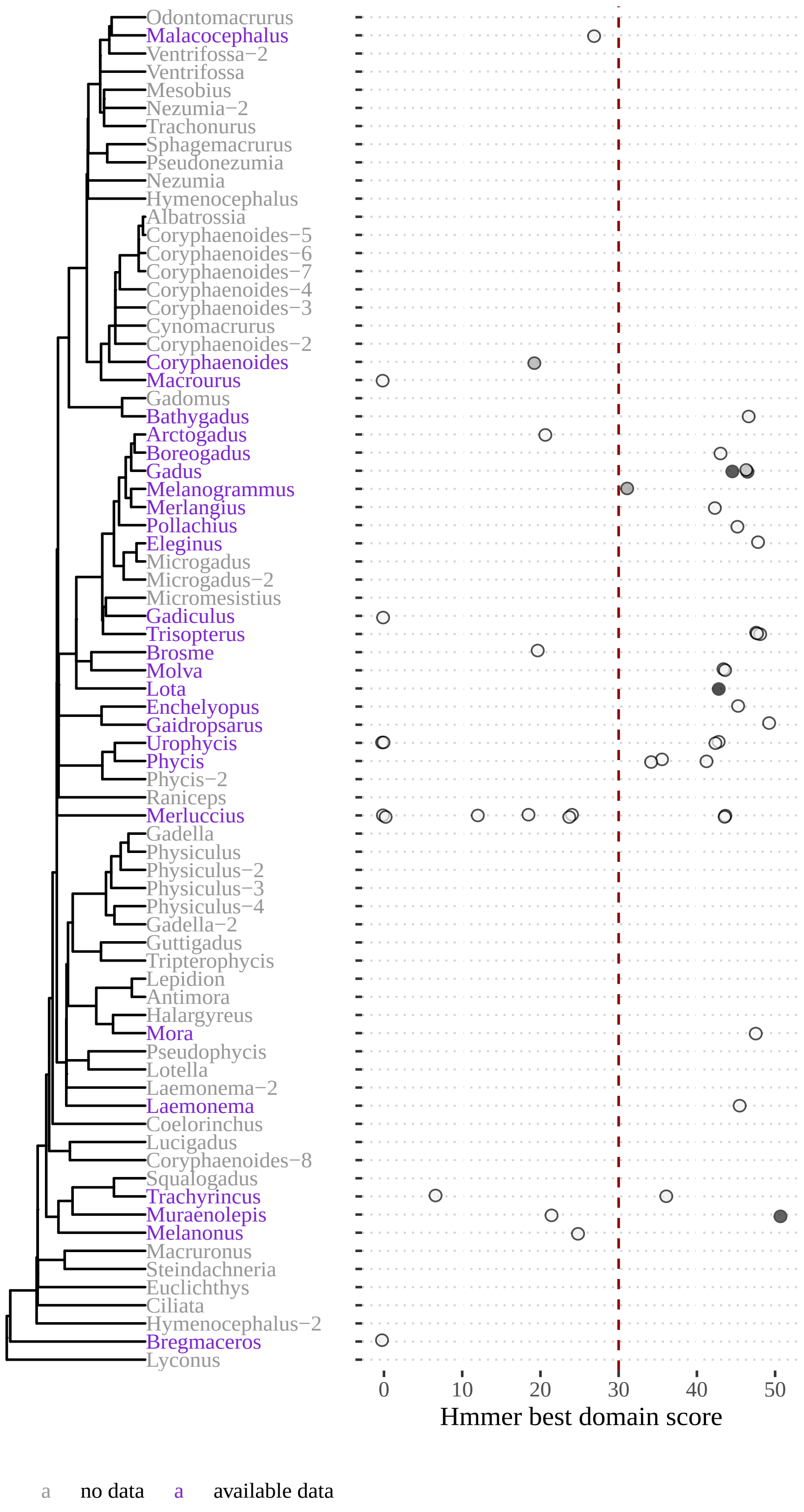

Fig. S18 aKRAB distribution in Gadiformes.
Phylogenetic tree of gadiform genera included in this study with associated hmmer best one domain score. Points are colored by $\log_{10}$ contig N50 of respective genome assembly. Some genera are represented multiple times on the tree (denoted by the '-' suffix), indicating alternative placements on the tree.

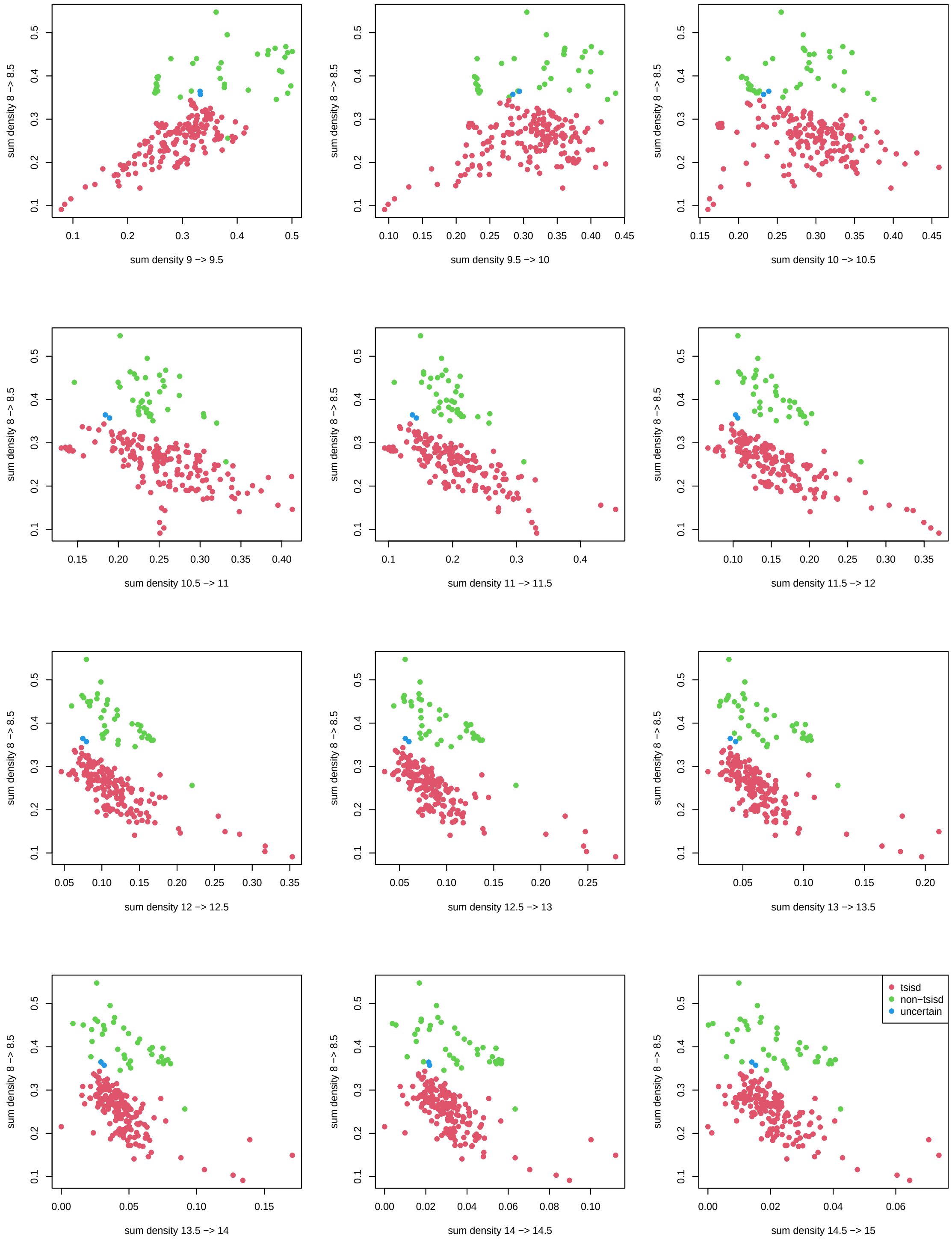

Fig. S19 Distinct distributions of teleost intron sizes.
$\log_2$ transformed density distributions of teleost intron sizes

References

[1] Yu, G., Smith, D.K., Zhu, H., Guan, Y., Lam, T.T.-Y.: Ggtree: An r package for visualization and annotation of phylogenetic trees with their covariates and other associated data. Methods in Ecology and Evolution **8**(1), 28–36 (2017) https://doi.org/10.1111/2041-210X.12628

[2] Wickham, H.: Ggplot2: Elegant Graphics for Data Analysis. Springer, New York, NY (2009). https://doi.org/10.1007/978-0-387-98141-3

[3] Kumar, S., Suleski, M., Craig, J.M., Kasprowicz, A.E., Sanderford, M., Li, M., Stecher, G., Hedges, S.B.: TimeTree 5: An Expanded Resource for Species Divergence Times. Molecular Biology and Evolution **39**(8), 174 (2022) https://doi.org/10.1093/molbev/msac174

[4] Pagès, H., Aboyoun, P., Gentleman, R., DebRoy, S.: Biostrings: Efficient Manipulation of Biological Strings. (2022). R package version 2.64.1. https://bioconductor.org/packages/Biostrings

[5] Jones, P., Binns, D., Chang, H.-Y., Fraser, M., Li, W., McAnulla, C., McWilliam, H., Maslen, J., Mitchell, A., Nuka, G., Pesseat, S., Quinn, A.F., Sangrador-Vegas, A., Scheremetjew, M., Yong, S.-Y., Lopez, R., Hunter, S.: InterProScan 5: Genome-scale protein function classification. Bioinformatics **30**(9), 1236–1240 (2014) https://doi.org/10.1093/bioinformatics/btu031

[6] Bodenhofer, U., Bonatesta, E., Horejs-Kainrath, C., Hochreiter, S.: msa: an r package for multiple sequence alignment. Bioinformatics **31**(24), 3997–3999 (2015) https://doi.org/10.1093/bioinformatics/btv494

[7] Lorenz, P., Steinbeck, F., Krause, L., Thiesen, H.-J.: The KRAB Domain of ZNF10 Guides the Identification of Specific Amino Acids That Transform the Ancestral KRAB-A-Related Domain Present in Human PRDM9 into a Canonical Modern KRAB-A Domain. International Journal of Molecular Sciences **23**(3), 1072 (2022) https://doi.org/10.3390/ijms23031072

[8] Camacho, C., Coulouris, G., Avagyan, V., Ma, N., Papadopoulos, J., Bealer, K., Madden, T.L.: Blast+: architecture and applications. BMC Bioinformatics **10**(1) (2009) https://doi.org/10.1186/1471-2105-10-421

[9] Paradis, E., Schliep, K.: ape 5.0: an environment for modern phylogenetics and evolutionary analyses in R. Bioinformatics **35**, 526–528 (2019) https://doi.org/10.1093/bioinformatics/bty633

[10] Eddy, S.R.: HMMER User's Guide

[11] Danecek, P., Bonfield, J.K., Liddle, J., Marshall, J., Ohan, V., Pollard, M.O., Whitwham, A., Keane, T., McCarthy, S.A., Davies, R.M., Li, H.: Twelve years of SAMtools and BCFtools. GigaScience **10**(2) (2021) https://doi.org/10.1093/gigascience/giab008 https://academic.oup.com/gigascience/article-pdf/10/2/giab008/36332246/giab008.pdf. giab008

[12] Smit, AFA., Hubley, R., Green, P.: RepeatMasker Open-4.0. 2013-2015. http://www.repeatmasker.org

[13] Gabriel, L., Brůna, T., Hoff, K.J., Ebel, M., Lomsadze, A., Borodovsky, M., Stanke, M.: BRAKER3: Fully automated genome annotation using RNA-Seq and protein evidence with GeneMark-ETP, AUGUSTUS and TSEBRA. bioRxiv: The Preprint Server for Biology, 2023–0610544449 (2023) https://doi.org/10.1101/2023.06.10.544449
